# A Tilted Axis: Maladaptive Inflammation and HPA Axis Dysfunction Contribute to Consequences of TBI

**DOI:** 10.3389/fneur.2019.00345

**Published:** 2019-04-24

**Authors:** Zoe M. Tapp, Jonathan P. Godbout, Olga N. Kokiko-Cochran

**Affiliations:** Department of Neuroscience, Institute for Behavioral Medicine Research, College of Medicine, The Ohio State University, Columbus, OH, United States

**Keywords:** traumatic brain injury, psychiatric disorders, neuroinflammation, microglia, stress, HPA axis, glucocorticoids

## Abstract

Each year approximately 1.7 million people sustain a traumatic brain injury (TBI) in the US alone. Associated with these head injuries is a high prevalence of neuropsychiatric symptoms including irritability, depression, and anxiety. Neuroinflammation, due in part to microglia, can worsen or even cause neuropsychiatric disorders after TBI. For example, mounting evidence demonstrates that microglia become “primed” or hyper-reactive with an exaggerated pro-inflammatory phenotype following multiple immune challenges. Microglial priming occurs after experimental TBI and correlates with the emergence of depressive-like behavior as well as cognitive dysfunction. Critically, immune challenges are various and include illness, aging, and stress. The collective influence of any combination of these immune challenges shapes the neuroimmune environment and the response to TBI. For example, stress reliably induces inflammation and could therefore be a gateway to altered neuropathology and behavioral decline following TBI. Given the increasing incidence of stress-related psychiatric disorders after TBI, the degree in which stress affects outcome is of particular interest. This review aims to highlight the role of the hypothalamic-pituitary-adrenal (HPA) axis as a key mediator of stress-immune pathway communication following TBI. We will first describe maladaptive neuroinflammation after TBI and how stress contributes to inflammation through both anti- and pro-inflammatory mechanisms. Clinical and experimental data describing HPA-axis dysfunction and consequences of altered stress responses after TBI will be discussed. Lastly, we will review common stress models used after TBI that could better elucidate the relationship between HPA axis dysfunction and maladaptive inflammation following TBI. Together, the studies described in this review suggest that HPA axis dysfunction after brain injury is prevalent and contributes to the dynamic nature of the neuroinflammatory response to brain injury. Experimental stressors that directly engage the HPA axis represent important areas for future research to better define the role of stress-immune pathways in mediating outcome following TBI.

## Introduction

More than 5.3 million individuals suffer from a traumatic brain injury (TBI) related disability in the US alone (1). The highest reported post-TBI impairment is the development or worsening of psychiatric disorders including depression, anxiety, and mood disorders, which can result in an overall decreased quality of life and increased long-term mortality (2, 3). For example, one study reported nearly half of patients suffering from a psychiatric disorder began experiencing symptoms only after TBI (4, 5). A major contributor to the development or worsening of psychiatric disorders after TBI is maladaptive inflammation and microglial priming (6, 7). Increasing evidence suggests that an altered response to stress can significantly compromise post-traumatic recovery and exacerbate the brain's already inflamed state, which can perpetuate long-term deleterious outcomes following TBI.

Broadly, stress is how the body reacts to changes in the environment in order to actively maintain homeostasis (8). Allostasis is the process by which homeostasis is maintained through change. Allostasis is accomplished through coordinated activation and regulation of the hypothalamic-pituitary-adrenal (HPA) axis, which releases stress hormones when activated. The primary stress hormone is cortisol in humans and corticosterone in rodents, both will be referred to as CORT in this review. CORT acts to regulate stress responses in the body and control other processes that maintain homeostatic balance, particularly inflammation and sleep. Dysregulation of the CORT response to stressful as well as non-stressful stimuli can have far-reaching effects on overall health and recovery from traumatic injury.

Clinical data indicate adrenal insufficiency due to suppressed activation of the HPA axis following TBI occurs in a quarter of all TBI cases (9). These clinical studies demonstrate that TBI induces baseline changes in neuroendocrine function but do not provide insight to if these changes influence post-injury response to and recovery from stressful stimuli, i.e., allostasis. Post-TBI HPA axis dysfunction leads to inappropriate responses to stress, which in turn can dysregulate inflammation. Both processes have been implicated in the development of psychiatric disorders after TBI; however, the cross-talk between stress-immune pathways after brain injury remains underexplored in both pre-clinical and clinical studies.

This review will report an overview of neuroinflammation after TBI, as well as a discussion on the inter-relationship between stress-immune pathways after brain injury. We will then discuss the clinical incidence of post-TBI HPA axis dysfunction and how experimental TBI models have similarly reported an altered stress response. Finally, we will discuss experimental approaches to characterizing post-TBI HPA axis dysfunction. Emphasis will be placed on the value of combination stress-TBI models that elucidate molecular mechanisms linking neuroendocrine impairment with immune dysregulation.

## Neuroinflammation After TBI

TBI occurs in two phases: primary and secondary injury. Primary injury is caused by mechanical forces of the injury itself and includes axonal shearing, hemorrhaging, and contusion (10). Primary injury occurs in varying degrees of severity often referred to as mild, moderate, or severe. There are many types of TBI which can be loosely categorized into diffuse and focal TBI. Examples of diffuse TBI are blast injuries or hypoxic-ischemic injury that has widespread damage but does not form a distinct lesion, while focal TBI includes skull fractures or foreign bodies such as a bullet or shrapnel that result in a lesion. There are many different experimental TBI animal models to represent both diffuse and focal TBI (11). A common model of diffuse TBI is fluid percussion injury (FPI), which is achieved through application of a fluid pulse to the intact dura mater of the brain (12). Controlled cortical impact (CCI) represents a focal TBI model caused by a piston applied directly to the brain, resulting in a contusion (13). Use of these varying types of injury models is vital to TBI research to develop precautionary measures and decrease the impact of different forms of primary injury.

Secondary injury is caused indirectly by TBI and results from prolonged processes that are initiated by trauma, including edema or changes in blood flow. Further, secondary injury constitutes neuronal damage and degeneration through molecular processes including mitochondrial dysfunction (14), oxidative damage (15), and neuroinflammation (16) making it a potential target for pharmacological intervention after TBI. Neuroinflammation is of particular interest in secondary injury because it has been implicated in the development of post-injury neurodegenerative disease (17, 18). As previously reviewed many cell types contribute to the neuroinflammatory response after TBI (19, 20), but much of pre-clinical and clinical research has focused on the role of the innate immune cell of the central nervous system (CNS), microglia. Microglial-mediated inflammation is associated with many symptoms after TBI including motor deficits (21), mood disorders (22), and neurodegeneration (23). Microglia thus provide a potential therapeutic target for ameliorating the negative effects of neuroinflammation as a part of secondary injury after TBI.

### Microglial-mediated Neuroinflammation After TBI Is Dynamic

Microglia are normally in a quiescent, surveying state that is characterized by small cell bodies and highly ramified processes. Acutely after TBI, numerous damage signals and cytokines such as IFN-γ or nuclear proteins from damaged cells engage microglia to induce an inflammatory response that can then persist to sub-acute and chronic time points. As the innate immune cell of the CNS, microglia have a conserved response to robust inflammatory stimuli such as a TBI. For example, following experimental midline FPI in rats, Iba-1 labeling shows that microglia are hypertrophied and de-ramified in the cortex and thalamus that persists to 7 and 28 days post-TBI, time courses that are considered sub-acute and chronic in experimental models of TBI, respectively (24). Antigen-presenting MHCII is highly expressed in these de-ramified microglia. These morphologically distinct microglia also have increased immunofluorescent expression of CD68, which is indicative of increased phagocytosis activity to clear debris from damaged and dead cells and promote healing after both diffuse and focal experimental TBI (25, 26). Both pre-clinical and clinical research characterize these de-ramified, MHCII^+^/CD68^+^ microglia as “reactive” and identify this pro-inflammatory state to mediate the rapid and robust neuroinflammatory response to TBI. Reactive microglia release pro-inflammatory cytokines such as IL-1, IL-6, and TNFα to perpetuate microglial-mediated inflammation and recruit other immune cells (27, 28). After experimental midline FPI in mice, *MhcII* and *Cd68* mRNA expression increases at 3 days post-injury and remains elevated at 1-week post-TBI (29). Reactive microglia promote a distinct profile of cortical inflammation, including temporal changes in inflammatory gene expression after midline FPI in mice (29). For example, gene expression of inflammatory chemokines including *Ccl7* and *Il-1*β is uniquely regulated at 8 h after TBI while toll-like receptor (TLR) genes, which participate in perpetuating pro-inflammatory signaling, are uniquely regulated 7 days post-TBI (29). The unique microglial profile at 8 h after injury is indicative of rapid increases in microglial reactivity in response to primary injury, while the microglial profile at 7 days post-TBI characterizes the persistence of secondary injury.

Experimental evidence has shown that this microglial reactivity persists chronically, indicating a mechanism by which inflammation may contribute to long-term consequences of TBI. After moderate experimental CCI in mice, reactive microglia reach their peak inflammatory state around 1 week post-injury, however remain reactive in the cortex, corpus callosum, and thalamus chronically after TBI, specifically at 52 weeks post-injury, as compared to sham controls (30). Further, this same study shows that chronic microglial reactivity correlates with neuronal loss as measured by increased lesion size. At 6 months and 1 year after experimental moderate CCI, the resulting lesion expands through the cortex and hippocampus, causing widespread neurodegeneration of the ipsilateral hemisphere. Collectively, these data illustrate the sub-acute and chronic responses of reactive microglia to TBI that not only perpetuate neuroinflammation, but also contribute to chronic neurodegeneration and expansion of the injury lesion. These findings are consistent with clinical analysis of post-mortem patient tissue samples from contusive, non-penetrating TBI that show microglial phagocytic activity is abundant at 15 and 30 days post-injury through increased CD68 labeling (31). Further, clinical evidence has shown that these reactive microglia persist chronically in patients for decades after a single TBI and can negatively influence cognitive function. One clinical study shows significantly higher microglial reactivity diffusely through the brain, particularly in the thalamus, via non-invasive PET imaging of PK binding in survivors of moderate to severe TBI as compared to age-matched controls (32). Though there is a large range of post-injury time-points−11 months to 17 years—each patient has persistent chronic inflammation that correlates with cognitive deficits. Patients' processing speed and response latency is significantly slower after TBI compared to age-matched controls and the severity of these cognitive deficits directly correlates with increased thalamic microglial reactivity. Notably, enhanced microglial reactivity is observed remotely from the site of injury and not observed around lesions from the TBI, which indicates widespread damage via secondary injury.

Reactive microglia are necessary to clear debris and modulate synapses immediately after TBI, but when chronically reactive can be neurotoxic. This is through perpetuating oxidative stress and neurotoxic cytokines such as nitric oxide (NO) via inducible nitric oxide synthase (iNOS) (33). NOX2, a reactive oxygen species (ROS), is highly expressed in the cortex at 1 and 2 days after experimental CCI in mice and co-localizes with morphologically reactive microglia (34). Another experimental study found that CCI in *Nox2*^−/−^ mice results in significantly reduced CD68 labeling, decreased lesion area, and reduced gene expression of inflammatory cytokines including *Il-1*β, *Il-6*, and *Tnf*α (35). This indicates that NOX2 is not only a cytotoxic product of microglial reactivity but can engage with other microglia to perpetuate microglial-mediated inflammation. Clinical analysis of human autopsy samples from fatal TBI cases also show significant increases in NOX2 expression throughout the cortex as compared to controls (36). Though transient microglial reactivity is necessary for appropriate immune responses to injury, persistent inflammation is maladaptive and can lead to neuronal degeneration and cell loss.

Experimental manipulation of microglia illustrates the dynamic role these CNS innate immune cells play in regulating inflammation following TBI. For example, reactive microglia present a potential therapeutic target of post-TBI inflammation, but research has shown the necessity of short-term microglia-mediated inflammation for recovery after models of experimental TBI. Activation of fractalkine receptor (CX3CR1) via neuronal fractalkine negatively regulates reactivity in microglia and results in a decreased pro-inflammatory phenotype. Following injury, affected neurons reduce release of fractalkine, resulting in increased microglial reactivity. To better understand the time course of CX3CR1 action after TBI, *Cx3cr1*^−/−^ mice were given experimental CCI and were tested on cognitive and sensorimotor abilities at 4 days and 5 weeks post-injury. At 4 days post-injury, *Cx3cr1*^−/−^ *mice* have improved sensorimotor function and decreased *Cd68* expression at 4 days after experimental CCI as compared to wild-type controls (37). This indicates that microglial-mediated inflammation acutely after TBI is protective against worsened outcome. Strikingly though, at 5 weeks after TBI *Cx3cr1*^−/−^ mice have reduced sensorimotor function and increased pathology shown by reduced neuronal count and increased microglial cell-body area as compared to wild-type mice after CCI. Thus, short-term inflammation due to increased microglial reactivity is necessary for recovery immediately following TBI however can be deleterious at chronic time points. Endogenous control of microglia, here being anti-inflammatory neuronal action, is needed for appropriate long-term recovery.

Another method of pharmacological manipulation to characterize effects of microglial reactivity after TBI is elimination and repopulation of microglia through antagonism of colony stimulating factor 1 receptor (CSF1R). CSF1R antagonists significantly deplete microglial populations in adult mouse brains *in vivo* after 7, 14, and 21 days of dosing through chow (38). Once CSF1R antagonism ceases, microglia begin to rapidly repopulate at 3 days post-treatment and return to pre-treatment levels of microglial population at 14 days. Many studies have used CSF1R antagonists in an attempt to replace reactive microglia with quiescent microglia. For example, *CaM/TetDT*_*A*_ mice express diphtheria toxin A chain (DT_A_) in forebrain excitatory and hippocampal CA1 neurons when doxycycline is not provided in their diet, leading to a lesion of neuronal death to mimic injury and disease (39). This same model previously reported robust microglial reactivity that resolves following microglial elimination (40). Once *CaM/TetDT*_*A*_ mice developed a lesion over 30 days, doxycycline was given to cease DT_A_ expression and after 7 days of recovery the CSF1R antagonist PLX5622 was given through chow for 14 days to eliminate microglia followed by 21 days of microglial repopulation. These repopulated microglia appear morphologically non-reactive, indicated by smaller somas and ramified processes compared to non-repopulated animals (41). Further, expression of inflammatory markers including *Cd68* is significantly decreased in lesioned animals after repopulation compared to lesioned animals with no repopulation, demonstrating a rescue of the reactive microglial population after neuronal death. Gene expression through RNA transcript NanoString analysis further shows a decrease of reactive microglia through reduced expression of inflammatory genes involved in IL-1 and iNOS signaling. Elevated plus maze further shows that microglial repopulation in lesioned animals rescues anxiety-like behavior to control levels and Morris water waze shows improved spatial memory as compared to lesioned animals. Thus, these repopulated microglia appear less reactive and contribute to improved behavioral outcome following neuronal death, further implicating reactive microglia as a potential therapeutic target for chronic inflammation. Notably, other glial-mediated inflammation through astrocytes is not affected by microglial repopulation and perpetuates the pro-inflammatory response and micro-environment. Thus, inflammation caused by neuronal death is not entirely dependent upon microglia and may continue even in the absence of microglial-mediated inflammation. In the context of TBI, microglial elimination using PLX5622 for 14 days prior to experimental midline FPI recapitulates the decrease in inflammatory gene expression through NanoString RNA analysis including *Il-1*β and *Nf*κ*b* signaling at 7 days after injury. Interestingly, pre-treatment of TBI with PLX5622 significantly attenuates astrocyte-related neuroinflammation, as determined through decreased GFAP immunofluorescent expression. Even with this astrocytic-mediated inflammation decreased, microglial elimination does not significantly improve numbers of ATF3^+^ cells or NeuN^+^ nuclei, indicators of neuronal survival, at 7 days post-injury (29). Together, these data indicate that elimination of microglia has robust effects on microglial-mediated inflammation, however fails to significantly affect other glial cells and neuronal death. Ultimately, manipulation of microglia demonstrates the dynamic role that microglia play in the inflammatory response acutely after injury and chronically during recovery from TBI.

### TBI Induces Microglial Priming That Contributes to Post-TBI Sequelae

After TBI, part of the reactive microglial population returns to a quiescent surveying state while another population takes on an intermediate phenotype. This population is characterized by increased baseline expression of *MhcII* and *Cd68* and a lower threshold to become hyper-reactive in response to a secondary insult (42). Due to the hyper-reactive inflammatory response to a secondary challenge, this intermediate population is referred to as “primed” microglia. Microglial priming was first described in a prion-disease animal model, ME7, subsequent to peripheral injection of the endotoxin, lipopolysaccharide (LPS) (43). After peripheral LPS challenge in ME7 animals, inflammatory cytokines including IL-1β, TNFα, and IL-6 are significantly elevated compared to control animals treated with LPS. LPS in ME7 animals also significantly increases the number of apoptotic cells in the hippocampus compared to control groups. This shows that exacerbated neuroinflammation due to microglial priming significantly increases neurotoxic effects of inflammation. Notably, LPS injection directly into the ventricles of ME7 animals does not result in the same hyper-reactive and enhanced apoptotic profiles seen after peripheral administration. This demonstrates that the primed microglia population is sensitive to peripheral immune challenges, thus illness due to infection can significantly increase neuroinflammation without direct infection of the brain itself. As previously reviewed, this same heightened response to peripheral immune stimuli is normally seen with aging (44). This includes increased levels of pro-inflammatory cytokines like IL-6 and IL-1β and decreased anti-inflammatory cytokines IL-4 and IL-10. Aged microglia are resistant to regulation by anti-inflammatory mechanisms and efficacy of antigen presentation decreases with age, resulting in increased expression of MHCII and a shift from adaptive to innate immunity. Together, these data indicate that excess inflammation caused by microglial priming can be additive to pre-existing conditions and worsen outcome.

Increased microglial priming has been associated with psychiatric disorders and could provide a mechanism through which TBI induces altered behavioral responses and post-TBI psychiatric sequelae. After experimental midline FPI, microglia have enhanced expression of *MhcII* at 30 days post-TBI, indicating a persistent and reactive population of primed microglia. *Il-1*β and *Tnf*α expression is significantly increased in response to peripheral LPS injection compared to controls (45). Additionally, after experimental midline FPI, peripheral LPS injection decreases social interaction at 24 h after injection compared to both sham controls and TBI animals that received saline, an indicator of increased sickness or depressive-like behavior. TBI animals that received LPS also spend more time immobile during tail-suspension test at 72 h after injection compared to all other groups, which again represents increased depressive-like behavior, mirroring clinical data that inflammation is associated with behavioral deficits. Primed microglia are seen in clinical studies of depressed patients after suicide, as seen by an increased ratio of de-ramified reactive microglia over quiescent ramified microglia compared to age-matched controls (46). Expression of MCP-1, a chemokine involved in monocyte recruitment, is also significantly higher in depressed patients, further depicting exacerbated inflammation related to microglial priming. This indicates a strong relationship between increased microglial inflammation via priming and psychiatric symptoms. Together, these data indicate that microglial priming not only causes exacerbated inflammatory responses to secondary immune challenges such as LPS, but may also contribute to compromised post-TBI behavior and psychiatric symptoms.

## Stress Influences Inflammation Through Glucocorticoids

**Figure 1:**
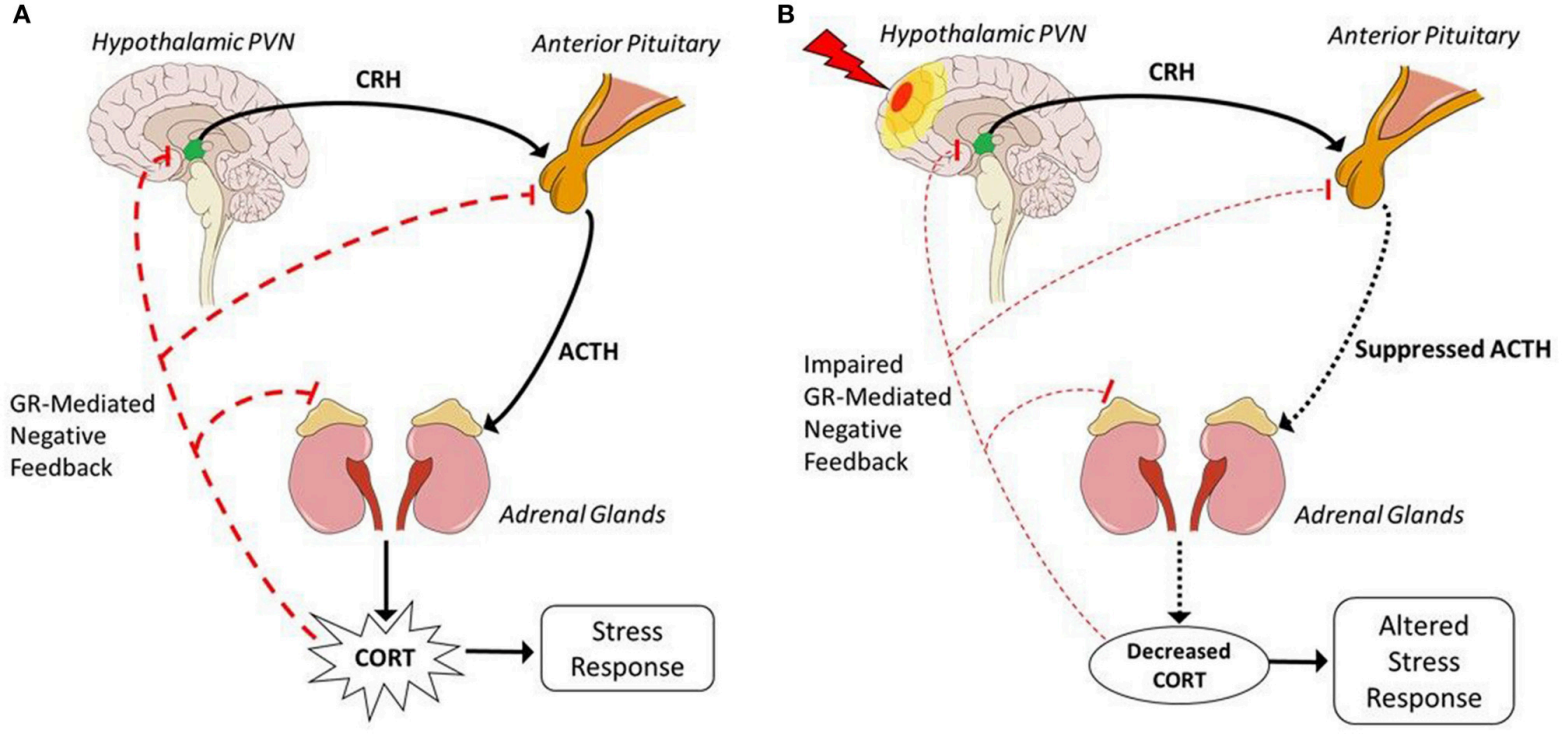
Hypothalamic-pituitary-adrenal axis before and after TBI. **(A)** In response to stressors, excitatory neuronal inputs activate the HPA axis and are transformed into hormonal communication, represented by solid black lines, to produce a physiological stress response. Activation via excitatory neuronal inputs to the hypothalamic PVN releases CRH and AVP via the median eminence into hypothalamic hypophyseal portal circuitry to the anterior pituitary. CRH induces corticotropes in the anterior pituitary to stimulate production of ACTH. ACTH is released into the blood and travels to the adrenal glands, superior to the kidneys. In the adrenal glands, ACTH initiates synthesis of CORT. CORT is then released into the blood to act on multiple tissues such as the lungs, heart, and muscles to induce a stress response. Represented by dashed red lines, CORT acts through GR-mediated feedback at every level to negatively regulate HPA activation and reduce CORT production. **(B)** TBI, represented as a lightning bolt, induces hypopituitarism and results in suppressed HPA activation in response to a stressor, represented by dotted black lines. Hypopituitarism indicates decreased production of ACTH, thus there is decreased stimulation of the adrenal glands and less CORT production. Suppressed CORT levels cannot inhibit continued HPA activation through GR-mediated negative feedback, as depicted by dashed red lines, resulting in impaired GR-mediated negative feedback and perpetuation of the stress response that leads to longer recovery time after exposure to a stressor. Decreased CORT is associated with increased inflammation which could contribute to psychiatric sequelae, thus injury-induced suppression of the HPA axis depicts a mechanism through which post-TBI consequences may occur. Sagittal brain schematic: Patrick J. Lynch, medical illustrator [CC BY 2.5 (https://creativecommons.org/licenses/by/2.5)].

Stress broadly refers to the disruption of homeostatic balance by a stressor. There are many different types of stressors (e.g., physical, psychological, immunological, etc.) that can result in a stress response. This stress response is characterized by activation of the HPA axis (Figure 1A). The neural and non-neural components of the HPA axis have many regulatory controls to ensure that the reactivity is appropriate for stressful as well as non-stressful stimuli such as sexual experience, appetite, or even light (47). HPA activation via stressful stimuli is a non-voluntary response tightly coupled to environmental events or cues that may disrupt homeostasis. Different types of stressful stimuli activate inputs to the hypothalamic paraventricular nucleus (PVN). Once activated, PVN neurons secrete corticotropin releasing hormone (CRH), also known as corticotropin releasing factor, and arginine vasopressin (AVP). These hormones then move to the “master gland,” the pituitary gland. In the anterior pituitary, CRH activates special endocrine cells called corticotropes that post-translationally modify proopiomelanocortin (POMC) to produce adrenocorticotropic hormone (ACTH). ACTH then travels through the blood to the adrenal glands, set above the kidneys. This interaction initiates steroidogenesis and release of glucocorticoids (GCs), specifically CORT. CORT then travels throughout the body via the blood and acts on many different tissues to restore homeostasis.

Basal GC secretion is regulated by high affinity mineralocorticoid receptor (MR) feedback inhibition, while regulation of GC responses to HPA axis activation is attributed to low-affinity glucocorticoid receptor (GR)-mediated negative feedback (48). GR has a low affinity for GCs and thus exerts inhibitory control when HPA activation results in significantly elevated GC levels and MR becomes saturated. GCs act on GR in the brain, pituitary, and adrenal glands and are thus able to control GC production at every level of the HPA axis. The hippocampus is an area of major regulation by GC action on neural GR as seen through lesion studies that show destruction of the hippocampus perpetuates HPA axis response to stressors (49). GCs also act on neural GR in the cingulate cortex to inhibit HPA axis activation through innervation of the PVN, as one study shows application of CORT to the cingulate cortex decreases plasma levels of both ACTH and CORT (50). In the anterior pituitary, GR-mediated feedback acts on corticotropes to prevent ACTH release (51), as one clinical study shows intravenous treatment with GR agonist rapidly inhibits ACTH and CORT responses to exogenous CRH stimulation (52). In the adrenal glands, GC production is not linearly related to ACTH application, but rather is slightly reduced (53). This indicates that dynamic levels of GC production within the adrenal glands acts through GR-mediated feedback inhibition on steroidogenesis of GCs. Regulation at the hypothalamus is more complex. GCs act on the PVN to regulate energy and fluid homeostasis in response to stressors (54). Genetic knock-out of GR in the PVN causes elevated basal CORT levels in response to stress, however does not alter circadian release of GCs (55), indicating limited inhibitory regulation through GR-mediated feedback at the PVN. *In situ* hybridization of rat brain tissue reveals that GR mRNA is highly expressed in another hypothalamic nucleus, the arcuate nucleus (ARC) (56). Application of the GR antagonist, eplerenone (EPN), at the PVN does not significantly increase plasma CORT levels until 80 min after administration while EPN at the ARC very rapidly increases CORT levels only 10 min after application (57). This data shows that GR-mediated negative feedback of multiple nuclei modulates HPA axis reactivity at the hypothalamus. Notably, examples of GR-mediated positive feedback of the HPA axis also exist in the brain. For example, as previously reviewed areas important for fear-learning upregulate HPA axis activation through feed-forward GC action on the amygdala, bed nucleus of the stria terminalis, and medial prefrontal cortex, resulting in increased CRH release (58). Together, these data indicate a sensitive and tightly regulated control of GR-mediated release of GCs and HPA axis reactivity that contributes to maintaining appropriate stress responses and homeostasis.

**Figure 2:**
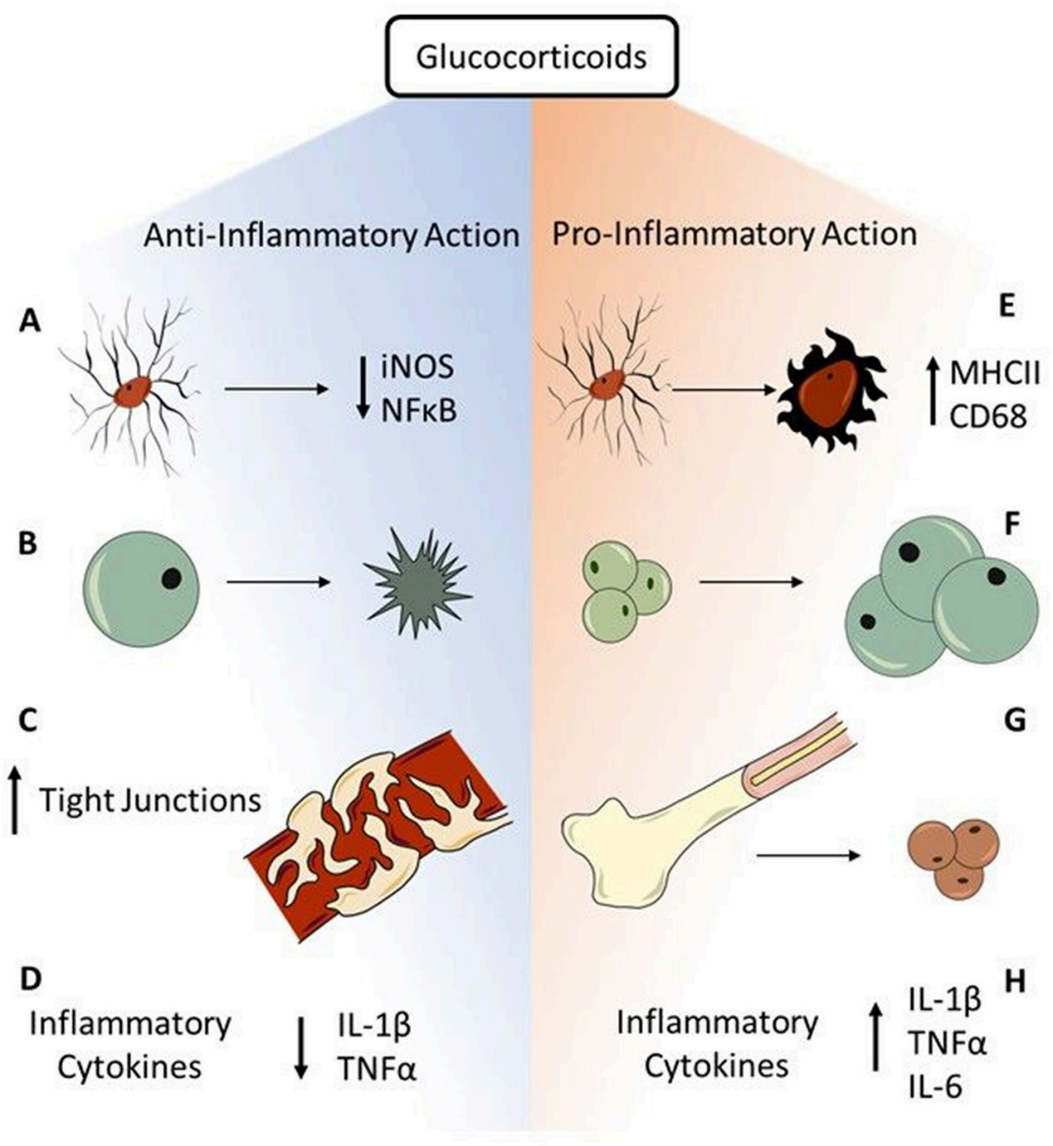
Anti- and pro-inflammatory actions of glucocorticoids. GCs have robust immunosuppressive function through multiple avenues in acute response to stressors. **(A)** Microglia have high expression of GR, making them prime targets for GC immunosuppression. GR-mediated GC action is neuroprotective through decreasing microglial iNOS synthesis and preventing NFκB activation. **(B)** The adaptive immunity effector cells, T-cells, go through GC-mediated apoptosis that prevents perpetuation of inflammatory responses. **(C)** Endothelial cells of the BBB express GR. Binding of GC to GR contributes to tightening of BBB tight junctions and improves BBB integrity to prevent peripheral immune cells from entering the brain. **(D)** The major anti-inflammatory mechanism of GCs is GR-mediated genomic immunosuppression through decreasing expression of inflammatory cytokines including IL-1β and TNFα. While GCs are primarily immunosuppressive in the face of acute challenges, chronic stressors reveal pro-inflammatory actions of GCs. **(E)** GR-rich microglia are susceptible to stress-induced priming, shown through increased CD68 and MHCII expression, resulting in a hyper-reactive response to subsequent immune challenge. **(F)** Though GCs induce T-cell apoptosis, GCs also enhance lymphocyte maturation through increasing T-cell sensitivity to IL-2, contributing to a pro-inflammatory environment. **(G)** GC production via the chronic stress of repeated social defeat mobilizes monocytes from the bone marrow, contributing to neuroinflammatory consequences such as increased anxiety. **(H)** GCs also increase circulating levels of IL-6, an inflammatory cytokine associated with trauma and illness, through activation of the HPA axis by multiple types of stressors. Further, chronic stress exacerbates the inflammatory profile through increasing expression of IL-1β and TNFα after excitotoxic cell death. Together, these anti- and pro-inflammatory actions exemplify the duality of GCs in mediating inflammatory responses to stress.

In addition to regulating the HPA axis, GR-mediated action of GCs plays a vital role in communication of the stress-immune axis and has many anti- and pro-inflammatory actions (Figure 2). As previously reviewed, GCs execute anti-inflammatory action through both genomic and non-genomic pathways (59, 60). GR-mediated anti-inflammatory action is primarily done through translocation of the GC-GR complex into the nucleus that binds to glucocorticoid responsive elements (GREs), for example to block the transcription factor, nuclear factor kappa B (NFκB)-mediated transcription of pro-inflammatory genes; non-genomic actions of GR have yet to be well-characterized (61, 62). Microglia integrate stress-immune interactions through high expression of GR (63), making them excellent targets for GC immunosuppression. For example, a co-culture experiment of rat microglia and neurons application of IFN-γ and LPS induced reactive microglia that are neurotoxic through oxidative stress via NO derived from inducible iNOS (64). Application of a synthetic GC, dexamethasone (DEX), is neuroprotective by significantly downregulating microglial NO production and iNOS mRNA expression. This decrease of oxidative stress via reactive microglia displays highly neuroprotective effects of GCs through anti-inflammatory action. Further, expression of iNOS is associated with activation of NFκB. In mouse dendritic cell lines, induced iNOS expression and NO production via NFκB activation is inhibited in a dose-dependent manner by pre-treatment with DEX (65). These data show that GCs can reduce oxidative stress both before and after inflammatory stimuli induce microglial reactivity, identifying GC anti-inflammatory action as potently neuroprotective. Other immune cells express GR that mediates GC action to decrease inflammation. For example, as previously reviewed GCs induce GR-mediated apoptosis of the adaptive immunity effector cells, T-cells (66). Further, GCs have protective effects on tissues that do not directly perpetuate immune responses, but can contribute to a pro-inflammatory environment such as the blood-brain barrier (BBB) (67). After treatment with DEX, immortalized mouse brain endothelial cells injured with *in vitro* blast injury have improved trans-endothelial electric resistance recovery and increased tight junction ZO-1 immunostaining compared to untreated cells (68). This illustrates that GCs play an important role in BBB recovery, which then decreases inflammation by preventing peripheral immune cells from migrating to the brain. The primary anti-inflammatory role of GCs is through the GR-mediated activation of GREs, which results in suppressed mRNA expression of pro-inflammatory cytokines including IL-1β, TNFα, and IL-6 while also enhancing expression of anti-inflammatory cytokines such as IL-10 and TGFβ (69). Together, these data illustrate the potent anti-inflammatory action of GCs through mediating immune cell functions.

With GC's intrinsic anti-inflammatory action, corticosteroids were one of the first anti-inflammatory drugs tested as pharmacological treatment for inflammation after injury (70). Efficacy of GCs after TBI is controversial due to varying effects on inflammation dependent upon time of administration. After experimental weight-drop TBI, immediate treatment with DEX decreases cell numbers positive for markers of microglial/macrophage inflammation (EMAP-II, P2X4R, AIF-1) at 24 and 48 h after injury (71). By days 4 and 6 after TBI, though, there is no significant difference in inflammatory marker quantification compared to saline controls. Though GCs decrease inflammation immediately following TBI, these effects are transient and in clinical trials could increase mortality. A large scale clinical trial quantifying death rate within 14 days after hospitalization and treatment for TBI shows that there is a significant increase in mortality after treatment with intravenous corticosteroids compared to placebo group (72). Additionally, previous reviews discuss the major barrier of GC treatment causing GC resistance or insensitivity, thus further decreasing efficacy of treatment (73). Together, these studies show that though endogenous GCs have potent anti-inflammatory effects, treatment with exogenous GCs is not effective in curtailing maladaptive inflammation after TBI.

### Altered GC Release Influences Inflammation

This tight relationship of the stress-immune axes is highly susceptible to dysfunction. Specifically, inappropriate stress responses through excessive HPA axis activation are shown to increase inflammation. GC release acts in a feedforward mechanism known as the glucocorticoid cascade hypothesis (74), which results in an unchecked stress response and subsequent inflammatory response. For example, excessive HPA axis activation after TBI influences neuroinflammation through stress-induced priming of microglia. As previously discussed microglia highly express GR and are thus susceptible to stress-induced priming in the presence of excessive GC levels. Experimental administration of exogenous CORT alone in rats does not modulate gene expression of inflammatory cytokines *Il-1*β, *Tnf*α, or *Il-6* in hippocampal microglia. However, when LPS is given as an immune challenge at 24 h after CORT administration, hippocampal microglia significantly increase inflammatory cytokine expression in a LPS-dose dependent manner compared to vehicle controls (75). This indicates that stress via exogenous GCs primes microglia to become hyper-reactive in response to subsequent LPS immune challenge. These same results are seen after increases in endogenous CORT through exposure to a stressor. Twenty Four hours after inescapable tail-shock, LPS injection significantly increases hippocampal CORT and IL-1β compared to vehicle controls (76). This same experiment further shows that pre-treatment with a GR antagonist, mifepristone (MIF), before inescapable tail-shock rescues the exacerbated responses in hippocampal CORT and IL-1β after LPS injection. This indicates that GCs act directly on microglial GR to cause stress-induced priming. In the context of brain injury, pre-treatment with MIF 48 h before experimental CCI significantly prevents loss of CA1 hippocampal neurons 24 h after TBI (77). Rescue of neuronal loss after TBI via GR antagonism indicates that GC signaling, as either initial or secondary challenge, contributes to maladaptive inflammation after injury. Further, increased neuronal survival in the hippocampus could improve learning, memory, and cognition as well as emotional regulation, which would improve psychiatric symptoms commonly seen after TBI. Pre-clinical systematic review shows that stressors including psychosocial stress significantly increase microglial reactivity in the hippocampus and prefrontal cortex of experimental rodent models, which then contributes to altered behavioral responses like psychiatric disorders (78). Together, these data indicate that GCs act through microglial GR to prime microglia and cause exacerbated inflammatory responses. This increased inflammatory response to stress may then lead to increased neuronal death and psychiatric complications following TBI, lending to compromised post-injury recovery and decreased quality of life.

As with microglia, GCs also have pro-inflammatory action on other immune cells. GC-mediated apoptosis is necessary for appropriate development of T-cells in the thymus. Further, in stimulated rat T-cell cultures, application of CORT significantly enhances lymphocyte proliferation through increasing T-cell sensitivity to IL-2, contributing to a pro-inflammatory environment (79). Chronic GCs also have marked pro-inflammatory actions on peripheral tissues, notably the bone marrow. Mice treated with adrenalectomy have significantly decreased circulating CORT and IL-6 after repeated social defeat (RSD) stressor compared to control groups, indicating an abolishment of the normal stress response (80). Though there is no difference in the percent of monocytes and granulocytes in the bone marrow between adrenalectomized mice and controls after stress, circulating levels of monocytes in the blood are significantly decreased in adrenalectomized mice. This indicates that adrenalectomy, thus abolishment of the appropriate stress response, prevents release of monocytes into the blood while not affecting monocyte production in the bone marrow. Immunofluorescent expression of a key chemokine in maintaining the retention of monocytes in the bone marrow, CXCL12, is significantly decreased in animals with intact adrenal glands after stress while there is no difference of CXCL12 expression in adrenalectomized animals compared to non-stressed sham controls. This indicates that CORT production via stressors directly influences retention of monocytes in the bone marrow, resulting in increased monocyte mobilization in response to stress. Most notably, the percentage of the macrophage population in the brain is significantly increased in animals with intact adrenal glands after RSD while adrenalectomy attenuates macrophage populations after RSD. This shows that CORT release in response to stressors, such as RSD, contributes to increased mobilization of monocytes into the blood and increased macrophage populations in the brain, resulting in a pro-inflammatory environment and neuroinflammatory consequences such as increased anxiety (81). Stress also contributes to neuronal death as pre-treatment with chronic stress levels of CORT exacerbates gene expression of *Il-1*β and *Tnf*α after kainic acid-induced excitotoxic cell death (82). This seems counterintuitive to the previously discussed anti-inflammatory action of GCs, however anti-inflammatory effects of GCs are seen with pre-treatment of low- to mid-range doses of GCs while high doses of GC perpetuate inflammation. These same pro-inflammatory stress effects are seen in clinical populations, as clinical systematic review of the immunological effects of psychological stress show significant increases in circulating inflammatory cytokines including IL-1β, IL-6, and TNFα up to 2 h after exposure to a stressor (83). Together, these data indicate that robust activation of the HPA axis increases inflammation through action on multiple tissues. While GCs have potent anti-inflammatory effects, excessive GC release in response to HPA axis activation can contribute to increased inflammation.

On the opposite side of the spectrum, inadequate GC production in response to stress results in increased inflammatory cytokines that are normally counter-acted by GC immunosuppressive action, also contributing to increased inflammation. GCs are released in response to stress to restore homeostasis, thus suppression of GC levels either basally or in response to stress can significantly increase inflammation. For example, mice infected with murine molluscum contagiosum virus after adrenalectomy have 80% mortality between 36 and 72 h after infection while non-infected adrenalectomized mice and infected mice with intact adrenal glands have long term survivals (84). Clinically, this unchecked inflammation is seen in stress disorders that present with suppressed release of GCs and often increased basal inflammation. For example, patients diagnosed with post-traumatic stress disorder (PTSD) present with low basal CORT levels but higher levels of circulating pro-inflammatory cytokines including IL-1β, TNFα, and IL-6 (85). Similarly, patients diagnosed with chronic fatigue syndrome have low urinary free CORT levels (86) and severity of symptoms is highly correlated with increased levels of ROS and IFN-γ (87). Together, these studies demonstrate that stress influences immune processes and dysregulation of the HPA axis results in maladaptive inflammation.

## HPA Axis Dysfunction After TBI

Immediately after TBI there is acute activation of the HPA axis due to the stress of the injury. Mild blast TBI in mice increases circulating CORT immediately following injury that peaks at 3 h after TBI compared to controls and returns to baseline by 5 h post-TBI (88). This same increase in HPA axis activation is seen in clinical populations as one study showed in the first 1–2 days following mild and moderate TBI there is a significant increase in circulating CORT levels compared to age-matched controls (89). This same clinical study shows that following severe TBI, baseline serum CORT levels decrease 1–3 days following TBI. Though this decrease in stress could diminish inflammatory responses long-term following injury, suppressed HPA axis activation could result in worsened outcome after more severe TBI.

Insufficient stress responses are delayed in mild to moderate TBI as compared to severe TBI. At 4 weeks after experimental lateral FPI there is a significantly blunted CORT response to restraint stress compared to sham controls (90). Differential effects of injury severity on HPA axis dysfunction are also seen in experimental models of TBI. For example, experimental mild CCI attenuates CORT responses to stressors at 7 and 21 days after injury compared to sham controls, but significantly increases CORT responses to stressors at 34 and 70 days post-TBI (91). Moderate CCI shows no difference compared to controls in CORT responses at 7 days after injury, however there is a significant decrease in CORT responses to stressors at 21 days that persists to 70 days post-TBI. The same variance in suppressed endocrine function is seen after clinical presentations of TBI. One clinical study showed that after severe TBI, 25–100% of patients display insufficient circulating levels of GCs in response to pharmacologically induced stress within 10 days of injury (92). After mild to moderate TBI, insufficient circulating GC levels are reported in 16–45% of individuals within a year after injury (93, 94). The actual incidence of suppressed HPA axis activation after TBI is unknown due to many factors. Many don't seek medical treatment after mild TBI, which implies that incidence of mild injury-induced endocrine dysfunction likely occurs in much higher frequency. Additionally, screening for endocrine abnormalities is not standard care of treatment for TBI and thus will often go undiagnosed. Even if suppressed HPA axis activation is suspected, diagnostic tools and thresholds for diagnosable dysfunction are not standardized and are instead set by individual hospitals and clinicians, resulting in variance of incidence reports (95). This dynamic and under-diagnosed dysregulation of CORT responses following all severities of TBI contributes to compromised post-TBI recovery and the development of long-term consequences following injury.

### Causes of Post-TBI HPA Axis Suppression

HPA axis dysfunction after TBI may occur at any level of the HPA axis. For example, suppressed GC synthesis and release can occur when the hypothalamus is compromised and attenuates the release of CRH by the PVN. After experimental midline FPI in rats there is increased hypothalamic neuronal process complexity at 1, 7, and 28 days post-injury measured by neuronal Golgi staining (96). These same injured animals exhibited HPA axis dysfunction after FPI. DEX suppression of the HPA axis through GR-mediated feedback showed no significant differences in plasma CORT levels of injured animals 48 h after treatment, while sham controls have significantly suppressed CORT. This blunted CORT response is most likely due to disrupted feedback inhibition as seen through abnormal DEX suppression results. Changes in hypothalamic neuronal complexity in conjunction with impaired GR-mediated negative feedback indicate a causal relationship of HPA axis dysfunction and alterations in HPA axis circuitry. Together, these data describe mechanisms by which the hypothalamus may be directly affected by injury to induce suppressed GC release and impaired negative feedback. As previously discussed, the hypothalamic arcuate nucleus plays an important role in mediating rapid negative feedback of the HPA axis to the PVN. CCI in mice induces hypertrophied astrocytes in the arcuate nucleus, indicating increased inflammation (97). Clinical imaging studies also reflect possible hypothalamic dysfunction as seen after pediatric injury in children aged 8–18 years with nearly 10% atrophy of the hypothalamus at 12 months post-injury as compared to age-matched controls (98). These inflammatory and morphological differences isolated from the injury site could negatively impact the ability of the arcuate to participate in rapid regulation of the PVN, resulting in inappropriate activation and control of stress responses.

The highest incidence of post-TBI HPA abnormalities is due to dysfunction in the anterior pituitary gland, resulting in decreased ACTH release and thus decreased CORT (Figure 1B). The pituitary is especially susceptible to TBI and injury-induced pressure changes or bleeding. Longitudinal clinical studies report 41% of TBI patients with abnormally high morning CORT levels at 10 days after injury (99). By 3 months, 32% had abnormally low morning CORT levels. This incidence remains relatively stable at 6 and 12 months with abnormally low CORT levels reported in 37 and 35% of patients, respectively. Dysfunction in the pituitary causes decreased release of ACTH, thus the adrenal glands synthesize and produce less GCs. In response to this decrease, the sensitivity of negative feedback is increased to compensate. As such, increased inhibition can further decrease GC release and cause altered response to stressors. Due to the complexity of direct, indirect, and feedback regulation at all levels of the HPA, a single mechanism by which ACTH production is decreased is still unknown.

Post-TBI adrenal gland complications are relatively rare as the first clinical case study was reported in 1997 (100). The patient was admitted for rehabilitation 1 month after TBI due to persistent weakness, fatigue, and nausea and only after showing no significant improvement with rehab was given an endocrine workup that revealed suppressed release of GCs after application of exogenous ACTH. Within a week of starting treatment with synthetic GCs, prednisone and fludrocortisone, symptoms of dizziness and lethargy significantly improved. While rare, cases of adrenal insufficiency via damage to the adrenal glands could be left undiagnosed due to significant overlap in symptoms with TBI alone. This complication is often seen in cases of multiple traumas to the body that could result in direct injury to the adrenal glands and most often cannot be directly attributed to TBI, thus it is commonly referred to as comorbidity rather than a direct symptom of TBI.

### Consequences of Post-TBI HPA Axis Dysfunction

Excessive activation of the HPA axis following TBI and its effect on post-TBI recovery has been well characterized (7, 101). For example, CORT levels reach their peak after midline FPI in mice at 30 min after injury and robust HPA axis activation persists as CRH mRNA expression increases 40% at 2 h after injury compared to sham controls (102). Inhibition of this increased HPA activation via CRH antagonist at 15 min and 2, 4, 6, and 8 h after injury significantly decreases lesion volume by 45% (103). This shows excessive activation of the HPA axis after TBI increases neuronal death, which in turn could influence neuroinflammation. As previously described, excessive GCs due to TBI also induce microglial priming and increase inflammatory cytokines in response to stress, resulting in maladaptive and chronic inflammation.

Inflammatory effects of HPA axis suppression after TBI are not well characterized, though decreased GC release has been identified as a risk factor for increased mortality and compromised recovery after TBI. Persistent attenuation of HPA axis activation after moderate TBI compared to mild TBI indicates a possible mechanism by which more severe TBI is associated with worsened long-term outcomes and development of disabilities. In a clinical comparison study of post-TBI CORT levels, patients that present with plasma CORT levels in the lowest quartile of the mean within 10 days of TBI have significantly higher mortality compared to all other quartiles (104). Further, patients that present with persistent CORT deficiency that lasts beyond 10 days post-TBI also have significantly higher mortality compared to those who present with transient CORT deficiency. Thus, depleted plasma CORT levels due to HPA axis dysfunction are significantly correlated with increased mortality following TBI. There is clinical evidence that insufficient ACTH production, which would contribute to suppressed levels of CORT, persists to 1 and 5 years after TBI as seen through abnormally low baseline serum ACTH and decreased responses to ACTH stimulation (105). Persistence of abnormal endocrine responses could contribute to elevated neuroinflammation via decreased anti-inflammatory action of GCs.

Age at the time of injury in adults does not seem to affect vulnerability to HPA axis dysfunction after TBI as most studies report no correlation with age (93). After pediatric TBI, though, children under the age of 12 are more susceptible to HPA dysfunction after injury as compared to older adolescents (106). In fact, one study found all surveyed children with mild to severe TBI have significantly suppressed CORT release in response to HPA activation through insulin tests (107). Injury acts as an early life stress exposure in pediatric TBI, which may induce maladaptive HPA axis maturation and thus permanently increase susceptibility to stress later in life (108). Combined with altered stress responses due to HPA axis dysfunction, pediatric TBI can significantly alter normal aging and contribute to increased chances of psychiatric sequelae and decreased quality of life. These data characterize a growing population of individuals who are aging with injuries and could be at higher risk of developing TBI-related disabilities due to age of injury and the persistence of an altered stress response.

HPA axis dysfunction is heavily implicated in the development or worsening of psychiatric symptoms after TBI. GCs regulate motivation and emotion through mediating actions on limbic circuitry (109). Major depressive disorder is characterized by HPA axis hyperactivity and dysregulated feedback inhibition, much like is seen acutely after mild TBI (110, 111). Review of post-TBI imaging studies shows significant differences in diffusion tensor imaging of many cortical areas such as the prefrontal cortex, ventral frontal lobe, and anterior temporal lobe (112). As previously reviewed, all these regions are associated with increased anxiety and depressive symptoms after TBI as they feed into the HPA axis and are heavily involved in mediating appropriate stress responses to emotional stressors (113). TBI-induced alterations in the circuitry of these sites results in compromised emotional regulation, contributing to many common symptoms after TBI such as irritability and frustration. Further, decreased CORT production causes symptoms such as fatigue, weakness, and weight loss. These same symptoms are commonly seen after TBI and broadly referred to as post-concussion syndrome (PCS). Other PCS symptoms include deficits in learning and memory, impaired focus, and irritability, which again reflect symptoms of HPA axis dysfunction. Case studies of suppressed CORT after TBI often describe patients with psychological sequelae thought to be associated with PCS because of this symptomatic overlap (100, 114). As the awareness of post-injury HPA axis dysfunction increases, it is now advised that endocrine testing be done throughout post-TBI recovery when symptoms that appear immediately after injury persist for 3 months or when delayed symptoms appear within 3 years after TBI (115). Interplay of abnormal neuroendocrine responses and compromised psychiatric behavior illustrates the importance of HPA axis dysfunction and long-term TBI recovery.

## Experimental Stress Models Provide Insight to the Relationship of Post-TBI HPA Axis Dysfunction, Inflammation, and Outcome

Chronic effects of altered stress responses after TBI on inflammation are poorly understood. Though altered stress responses are increasingly acknowledged as an important clinical consequence of TBI, the mechanism by which HPA axis dysfunction contributes to post-injury symptoms has yet to be identified. One of the best resources for long-term outcome after TBI is clinical surveys, which can be restricted by lack of specific medical history and experience. As previously discussed, clinical surveys can also be affected by low reporting numbers or differences in endocrine testing, resulting in variable numbers and diagnostic tools. Experimental models of stress are necessary to better characterize chronic effects of stress after TBI and resulting inflammation. Currently, many studies have illustrated altered stress responses after experimental TBI, but few studies have looked at the consequent effects of experimental stress on inflammation after TBI. Here, we describe common stress models and further highlight combination studies in which stress models characterize HPA axis dysfunction after experimental TBI. Further, we discuss evidence that these stress models influence inflammation and could thus characterize stress effects on inflammation after TBI. While “stress” can be induced by a variety of stimuli, these established stress models are repeatedly used due to their efficacy in best illustrating the HPA axis response.

### Restraint Stress

The most common model for characterizing stress effects after TBI is restraint-induced stress. Restraint stress uses the inescapable stress of immobilization to induce robust HPA activation and GC release. After 6 h of daily restraint for 28 days, plasma CORT levels of stressed mice are significantly increased during the stressed period compared to non-stressed controls, however these effects do not persist at 2 or 4 weeks after daily stress concludes (116). Notably, though, prolonged restraint stress induces depressive-like behavior as seen through increased immobility during forced swim task throughout the 28 days of daily stress exposure, which persists 1 week after restraint stress ceases. Restraint stress thus shows that though robust prolonged stress may not cause persistent alterations in CORT levels, behavioral effects of stress persist even once the stressor has ceased. Further, 10 days of daily 2-h restraint stress in mice significantly increases inflammatory gene expression including IL1A, IL6, and TNFα compared to non-stressed controls (117). These inflammatory effects of restraint stress could contribute to post-stress behavioral deficits and could be further exacerbated by injury. Restraint stress after experimental TBI causes suppressed CORT responses to stress like that seen in clinical populations after TBI. Midline FPI chronically reduces basal CORT levels at 54 days post-TBI compared to sham control animals and baseline CORT levels before TBI was given (96). In response to 30 min of restraint stress, injured animals have decreased plasma CORT levels 1 h after restraint stress has concluded compared to sham controls. These data show that restraint stress is an efficient stress model that specifically and robustly activates the HPA axis, influences inflammation, and characterizes endocrine dysfunction after TBI.

### Forced Exercise

Another model of chronic stress after TBI is forced exercise. Forced exercise consists of an enclosed treadmill or wheel that is set at a specific speed with either prodding or small foot shocks to ensure the animal keeps pace. Compared to 1 h of spontaneous wheel exercise, 1 h of forced wheel exercise significantly increases CRH mRNA expression in the PVN (118). Increased CRH indicates substantial HPA axis activation specifically via forced exercise vs. voluntary exercise. In another experimental study, 30 min of forced treadmill exercise 3 times a week for 4.5 weeks significantly increases plasma CORT levels during open-field task compared to non-exercised controls (119). Additionally, elevated plasma CORT levels in animals exposed to forced exercise are correlated with increased IL-1β levels in the brain and decreased hippocampal neurons. This shows that forced exercise increases CORT through HPA activation and can influence inflammation and cell survival. In another experiment, animals were injured with mild/moderate FPI then exposed to two daily 20-min periods of forced running 1–4 and 7–11 days after injury while control animals either did not have access to a wheel or had access to a wheel for voluntary exercise (120). After forced exercise, ACTH and CORT levels are significantly elevated in both sham and injured forced exercise groups compared to voluntary exercise and sedentary controls. Notably, though, ACTH responses are blunted in the FPI groups. Additionally, forced exercise causes a decrease in hippocampal GR in sham injured animals compared to voluntary and sedentary groups; however, there are no differences in hippocampal GR between any groups after FPI. A lack of GR modulation in response to elevated CORT levels after FPI depicts a mechanism through which GR-mediate negative feedback is compromised after TBI. These data show that forced exercise is an effective stressor that increases inflammation and demonstrates injury-induced suppression of the HPA axis.

### Forced Swim

Forced swim is a common behavioral test, however it is also frequently used as a stressor to induce robust CORT responses (121–123). Forced swim involves placing an animal in an inescapable container of water deep enough that the animal cannot touch the bottom for a pre-determined amount of time. This acts as a behavioral test through measuring time immobile as an indicator of depressive-like behavior. The use of forced swim has shifted from a behavioral test to a stressor due to evidence that coping processes during forced swim are more indicative of stress-induced immobility rather than depressive-like behavior (124). There is a robust stress response in mice after forced swim stress for 1, 5, and 10 days of daily 30-min exposure as seen through significantly elevated levels of CORT compared to baseline CORT levels 60 min and immediately before the stress (125). Additionally, 5 days of daily 30-min forced swim stress increases IL-1β expression in the PVN and IL-6 expression in the hippocampus, indicating an increased inflammatory response to the stress of forced swimming. After mild and moderate CCI in rats, 15-min of forced swim at 21 and 54 days after injury significantly increases CORT responses in animals with mild injury while animals with moderate injury have blunted CORT responses (91). Forced swim is an effective stressor through HPA axis activation, increases pro-inflammatory profiles, and notably shows differential effects of injury severity on CORT responses.

### Foot Shock

The foot shock fear-conditioning paradigm traditionally tests anxiety-like behavior and conditioned fear memory. Animals are trained to associate a conditioned stimulus, such as a tone, with a foot shock. After training, animals' freezing behavior is measured in response to the conditioned stimulus. The HPA axis modulates fear conditioning through inducing CORT release that then acts on the hippocampus to increase contextual fear conditioning memory consolidation. Increased magnitude of foot shock is correlated with increased CORT release, which results in exacerbated behavioral deficits (126). Foot shock also increases inflammatory responses specifically in the HPA axis as 80 episodes of inescapable foot shock over the course of 2 h increases IL-1 gene expression in the hypothalamus and IL-6 in the pituitary (127). After lateral FPI, freezing behavior of injured rats increases in response to a conditioned tone as well as the context of the training chamber compared to sham controls in response to a strong shock paired stimulus as opposed to a weak shock (128). Increased freezing after foot shock training is indicative of anxiety-like behavior, illustrating behavioral deficits because of TBI. Further, western blot analysis shows that NR1, a marker of excitatory NMDA receptors, is significantly increased in the basolateral amygdala (BLA) and there is a trend of decreased GAD, a marker of GABA-ergic inhibitory neurons, in the BLA and hippocampus after FPI compared to shams. These alterations in excitatory and inhibitory input to important areas of emotional regulation further shows how TBI may affect psychiatric health and behavioral deficits. These data illustrate that foot shock not only acts as an effective measurement of post-TBI consequences, but also activates the HPA axis and increases stress-related inflammation.

### Stress Models Consequently Affect Sleep

Increased stress is both clinically and experimentally proven to induce sleep disturbances, even once exposure to a stressor has ceased (129). Coordination of GC release via HPA axis activation and environmental cues is vital for the conservation of normal sleep quality and longevity. Differential HPA activity mediates stages of sleep. Humans on average sleep 7 to 8 h at night with a sleep bout of 6 to 8 h and experience about 6 cycles of non-REM sleep (i.e., slow-wave sleep) and REM sleep (i.e., paradoxical sleep) (130). Mice, on the other hand, sleep for 12 to 14 h during the light phase with a sleep bout of only 2 to 4 min. Further, they have polyphasic sleep meaning that they experience cycles of non-REM and REM sleep periodically with waking period in between. Despite these differences in sleep architecture, features of rodent sleep models such as circadian and homeostatic modulation of sleep are similar to humans (131) and thus are often used in translational research.

At the onset of sleep, the HPA axis is suppressed to achieve minimal arousal. HPA axis activity increases along with sympathetic and brain activity during REM sleep and is once again suppressed as REM sleep concludes. As waking approaches, ACTH is released by HPA axis activation and causes increased arousal, concluding sleep (129). The HPA axis and sleep have a highly integrated relationship of both excitatory and inhibitory effects that depend upon the level of the HPA axis participating in regulation and phase of sleep. HPA axis control of sleep differs between CRH, ACTH, and CORT and at times seems contradictory. Continuous administration of CRH does not significantly alter non-REM and REM sleep, but continuous administration ACTH decreases time spent in REM sleep. Meanwhile, CORT both decreases time spent in REM sleep and increases non-REM sleep (132). Other studies have found CRH and CORT promote REM sleep (133). This contradictory relationship lends to CRH as a direct regulator of waking and arousal because it is directly controlled by suprachiasmatic nucleus “pace-maker” activity. GCs only indirectly affect waking through their inhibitory feedback action on CRH production (134). In REM sleep, exact roles of CRH or CORT remain unknown as studies report contradictory results of CRH not affecting or increasing REM sleep while CORT is reported to both enhance and decrease time spent in REM (135). Though specific effects of HPA axis activity on sleep remain controversial, the highly integrated and dynamic relationship between sleep and stress indicates an avenue by which models of stress influence sleep in experimental research. Restraint stress, forced exercise, forced swim, and foot shock all act as stress models through their ability to induce rapid HPA axis responses that cause robust spikes in CORT release. Interestingly, these models are also highly associated with sleep disturbances. For instance, exposure to 2 h of restraint stress during the beginning of the dark phase induces sleep rebound illustrated by increased REM sleep for the remaining 10 h of the dark phase (136). In comparison, gentle handling to induce sleep disruption does not result sleep rebound, demonstrating restraint stress-induced sleep disturbances and sleep rebound is not due to loss of sleep during the 2 h and is instead a consequence of the stressor itself. The specific effects of stress upon sleep can vary depending on many variables including time of day, strength of the stressor, metabolic state, and sleep need. With this, different amounts and types of stressors may influence non-REM and REM sleep in contradictory ways. Overall, though, alterations in the sleep/wake cycle as a result of stress can have long-lasting effects on behavior and cognition.

As with HPA axis activation influencing sleep, complex regulatory action of sleep upon HPA axis function influences stress responses. Sleep traditionally has suppressive effects on the HPA axis to maintain healthy and consistent sleep behavior before HPA activation induces waking behavior (137). When sleep itself is altered, the inhibitory control of the HPA axis during sleep can lead to hyperactivity in the stress response. Indeed, clinical sleep studies show that secretory profiles of CORT are influenced by both provoked and spontaneous awakenings in humans (138). CORT responses to spontaneous awakenings illustrate the intersection of stress-sleep pathways and how sleep disturbances may affect the stress response. Hyperactivity in the stress response due to decreased inhibitory control from sleep can increase negative feedback action by GCs, which could then further influence the stress response. Thus, a vicious cycle is born of the tightly coupled relationship between sleep and stress that concurrently and persistently is dysregulated. Experimental forced exercise stress model causes sleep disturbances through forcing the animal to remain awake and active, thus depriving sleep. Forced exercise on a treadmill or wheel for 24 h causes total sleep deprivation, abolishing both non-REM and REM sleep, and significantly elevates heart rate and body temperature, indicators of increased HPA axis activation (139). Stress response and performance in experimental forced swim stress model is influenced by circadian timing. For example, rats in nocturnal conditions display less escape behavior compared to those in diurnal conditions over the course of 5 min of forced swim (140). These behavioral differences reflect changes in serum CORT, which increase during nocturnal exposure to forced swim compared to diurnal conditions. Foot shock stress model influences sleep primarily by conditioned context. For example, at 24 h after exposure to foot shock fear conditioning, rats that sleep in conditioned context environments have selective decreases in REM sleep compared to animals that sleep in neutral context environments (141). Due to the interplay of sleep and stress axes, particularly regarding circadian release of CORT, alterations in sleep due to exposure to a stressor should be considered in the effect of stress on post-TBI outcome.

Many sleep disruption or deprivation models abolish REM sleep by taking advantage of stress influences on sleep. The elevated platform model, colloquially known as the “flower pot” model, uses a single small platform—such as an inverted flower pot—in a pool of water to selectively inhibit REM sleep (142). The animal can sustain non-REM sleep, but the loss of muscle tone in REM sleep causes the animal to fall into the water. This method thus employs fear conditioning to cause multiple awakenings throughout REM sleep, abolishing it completely. After 72 h of exposure to the elevated platform model, rats have elevated circulating CORT levels and body temperature that persist 7 days after REM sleep deprivation (143). A caveat of this restrictive platform model is that the single platform could induce a stress response due to restricted movement and social isolation, not just due to abolished REM sleep. Thus, a multiple platform model was developed which consists of numerous platforms placed in water to allow less restricted movement and multiple animals to be tested at one time while still not allowing enough consistent space for REM sleep (142). Efficacy of the multiple platforms test is controversial, though, as one study shows housing of multiple rats during the multiple platform method causes forced wakefulness through social interaction and decreases number of sleep bouts compared to the single platform method (144). Stress-free sleep models ensure sleep is disrupted through manipulation of sleep alone and not through stress-induced sleep loss. Manual handling is a common stress-free sleep model where an experimenter gently handles animals to prevent sleep (145). Though efficient in preventing sleep, manual handling may be inconsistent between experimenters and there is evidence that suggests mice have olfactory-induced HPA activation in response to male investigators compared to female investigators during gentle handling (146). Automatic sleep disruption models use mechanical disruptors such as air puffs or sweeper bars to wake animals without experimenter intervention. The air puff method selectively inhibits REM sleep through EEG and EMG surveillance of the animal's sleep state. When an animal is entering REM sleep the system automatically releases a puff of air to disturb the animal and disrupt REM sleep (147). Alternatively, the air puff method can disrupt sleep at predetermined intervals to globally disrupt both non-REM and REM sleep. An automatic sweeper bar similarly disrupts sleep by forcing animals to awake and move over a mechanical bar that travels the length of the cage (148). As with the air puff method, this can be done to either selectively eliminate REM sleep in response to EEG and EMG signals or globally disrupt sleep through pre-set intervals. Automatic sleep disruption systems ensure that sleep disturbances are modulating HPA axis reactivity and inflammatory profiles without the presence of excess stressors.

### Sleep Disruption Worsens Outcome After TBI

Sleep disorders including insomnia, hypersomnia, and sleep apnea occur in 30–70% of those who suffer from TBI regardless of severity (149). This is such a large range because much like HPA axis dysfunction, the extent to which sleep disturbances and disorders affect those after TBI is often underreported. Daytime sleepiness and hypersomnia are among the most commonly reported sleep disturbances acutely after injury, however opposing disorders such as insomnia have been reported in anywhere from 5 to 25% of people after TBI (149). Injury-induced alterations in sleep are also seen in experimental models of TBI. Midline FPI increases overall sleep during the first 6 h after injury compared to sham controls (150). This increased sleep need defines a period of vulnerability to a second TBI as mice injured again with midline FPI during this period at 3 h post-injury have increased motor deficits, neurological severity scores, anxiety-like behaviors, and neuropathology compared to mice given a second injury at 9 h post-injury. The extent of this sleep need is limited and increased sleep need persists to 7 days after midline FPI in mice but quickly resolves with no significant sleep disturbances as a result of TBI at 2 to 5 weeks after injury (151). Though experimental models of TBI fail to recapitulate chronic deficits in sleep/wake behavior, they still contribute to identifying candidate mechanisms for translation to humans. For example, hypocretin, also known as orexin, primarily promotes wakefulness behavior and is implicated in regulating reward, emotion, and stress circuitry (152). One study found that hypocretin may mediate sleep/wake following TBI through us of a hypocretin knock out (HCRT KO) mouse model (153). Following moderate CCI, HCRT KO mice showed no altered sleep/wake behavior, while wild-type mice exposed to the same injury had more non-REM sleep and less wakefulness. For example, mice exposed to a blast-wave TBI show long-lasting DNA methylation differences at 8 months post-injury in important regulatory genes for neuronal survival and inflammation such as TGFβ signaling, iNOS, and IL-1β (154). Notably, methylation differs in important circadian rhythm genes including period circadian clock 3 (*Per*3), a regulator of circadian rhythm, and *Aanat*, which codes an enzyme involved in conversion of serotonin to melatonin. Though sleep disturbances were not measured in these animals, long-term differences in DNA methylation of circadian genes indicates a possible epigenetic modulation of sleep through TBI.

Sleep disruption models, especially those that modulate HPA axis activation through sleep disturbances alone, are necessary to explore the effects of sleep/wake disruption after TBI as a unique stressor that engages the HPA axis. Sleep disruption also influences inflammation, and thus is a physiologically relevant stress model to further characterize post-TBI effects on stress and immune axes. Circadian release of GCs influences sleep onset and completion. Consequently, sleep can decrease or increase HPA axis activation and reactivity in response to stressors. Clinically, sleep loss increases the expression of key inflammatory cytokines that are also involved in the neuroinflammatory response such as IL-1, IL-6, and TNFα (155). Clinical surveys of post-TBI sleep disruption are numerous, but few experimental studies have investigated how altered sleep can influence processes such as the HPA axis and inflammation to affect post-TBI outcomes, and instead show no broad outcomes of post-injury sleep disruption. For example, one experimental study reports that 48 h of total sleep deprivation using forced exercise in rats does not increase neuronal susceptibility to mild TBI (156). In another study, 6 h of gentle handling sleep disruption immediately following midline FPI in mice shows no significant differences compared to non-sleep disrupted mice in circulating CORT levels, neurological severity score, or novel-object exploration at 1, 3, 5, or 7 days post-injury (157). Though limited reports show that acute sleep disturbances may not affect post-TBI recovery in experimental models, the effects of chronic sleep disruption remain unclear. Persistent sleep disturbances after injury could indicate a mechanism by which those who suffer TBI are more vulnerable to exacerbated stress and inflammation.

Sleep disruption can influence psychiatric symptoms and mood disorders as a result of TBI. Of nearly 50,000 military members that screened positive for blast-related TBI, sleep was found to mediate 26% of TBI's effect on PTSD and 41% of TBI's effect on depression (158). Clinically, presence of sleep disorders—specifically insomnia—reduces community integration following TBI and acts as either a mediator or contributor to depression through lowering quality of life (159). Also, sleep disturbances have been heavily implicated in either mediating or contributing to post-TBI sequelae such as PTSD (160), pain (161), and psychiatric disorders (162). For example, self-reports of depression and poor sleep quality are significantly associated with fMRI changes in the lateral orbitofrontal, dorsolateral prefrontal, and cingulate cortices (163). Moreover, cortical MRI analysis of post-acute period TBI patients consistently identifies connectivity and volumetric changes in the frontal and cingulate cortices as compared to age- and sex-matched controls (164, 165). Collectively, these data indicate that damage-induced alterations of these cortical areas could contribute to sleep-mediated mood disorders. As with HPA axis dysfunction, children are highly susceptible to post-TBI sleep disruption. Experimental models of pediatric TBI also show chronic sleep alterations. After repetitive experimental closed head TBI in 3-week-old mice, environmental enrichment to 11 weeks of age increased REM sleep in control mice while there was no effect of environmental enrichment after TBI (166). Sleep represents a highly translatable mechanism by which TBI can negatively impact vital processes to maintain homeostasis. Following pediatric injury in children aged 11–17 years, up to 67% report trouble with sleep onset, fatigue, and drowsiness immediately following injury and up to 38% report sleep problems persisting to 2–3 weeks after injury (167). Some studies have shown chronic persistence of sleep disorders in children independent of injury severity with reported sleep disturbances still experienced up to 2 years after injury (168). The likelihood of developing or worsening psychiatric disorders increases after pediatric TBI due to susceptibility to post-injury complications such as HPA axis dysfunction and sleep disturbances (169). Post-TBI psychiatric disorders as a symptom or result of the interplay between psychiatric dysfunction, altered sleep, and injury illustrates the importance of understanding the impact of TBI on homeostatic and allostatic systems.

## Future Steps in Pre-Clinical and Clinical Trauma Research

This review highlights topics that should continue to be considered in future pre-clinical trauma research. First, the temporal progression of central and peripheral immune response to TBI is dynamic and recovery is influenced by previous, concurrent, and subsequent immune challenge. This experimental trajectory is highlighted by previously discussed data that delineates the intersection of TBI and stress systems necessary to restore homeostasis after injury. Second, chronic post-injury time points are required to define the long-term consequences of brain injury. As previously discussed, the presence of HPA axis dysfunction has been identified in experimental models of TBI and corresponds with injury severity. Therefore, therapeutic interventions that intersect stress-immune pathways may require specific dosing strategies at acute and chronic post-injury time points. Third, there has been a dramatic surge in the development of pre-clinical TBI models. This is largely due to increased appreciation of brain injury after exposure to an explosive blast or repetitive concussion. These models are necessary to improve translation; however, much work is needed in identifying model specific stress-immune responses. Along with rodent models, much work has been done to better identify similarities and caveats of other animal injury models. Notably, large animal models such as pigs may offer additional insight due to the similar size, organization, and development of the brain to humans (170). Lastly, continued emphasis must be placed on translation from pre-clinical to clinical work while maintaining an appreciation for species-specific differences.

In one clinical survey, nearly a quarter of participants reported a lifetime history of TBI, which was associated with decreased quality of life (171). Aspects that contributed to this decreased quality of life include disability and lower incomes as well as increased risk of chronic poor health and more days of limited activities. Notably, participants also reported an average of less than 7 h of sleep per night. Work and health status are significant stressors when recovering and living with a TBI. These environmental stressors can further compromise post-injury recovery. There are many topics for consideration in clinical brain injury work to further advance TBI treatment. First, much of clinical research has focused on improving diagnosis, often through biomarkers (172), and immediate treatment (173) of TBI. Much like pre-clinical work, though, emphasis on understanding chronic effects of TBI will provide needed insight to unique immune and stress responses influencing recovery. Second, long-term clinical observation of homeostatic and allostatic systems is necessary for appropriate chronic treatment of patients with TBI. This could be addressed through standardizing the assessment of post-TBI HPA dysfunction at both acute and chronic post-injury time points. (174). Finally, acknowledging the need for potentially distinct acute and chronic therapeutic interventions may broaden the ability to manage temporal changes in stress-immune recovery after TBI.

Inflammation and stress processes are altered both acutely and chronically after TBI. Microglial priming contributes to exacerbated neuroinflammation, neuronal degeneration, and behavioral deficits following TBI. Substantial evidence, as reviewed here, indicates that HPA axis dysfunction after TBI is an under-appreciated but serious post-injury complication that can drastically affect the body's ability to maintain homeostasis and regulate the neuroinflammatory environment. Appropriate responses to environmental cues through GC production and release are required for basal inflammatory and stress responses. Long-term effects of TBI-induced HPA axis dysfunction on inflammation are currently unknown, but the stress-immune axis breakdown after injury could contribute to chronic inflammation, the development or worsening of psychiatric disorders, and altered behavioral responses. Application of stress models after experimental TBI could better elucidate a mechanism by which post-injury inflammation can both modulate and be modulated by HPA axis dysfunction. Particularly in response to changes in sleep/wake disturbances, HPA axis dysfunction following TBI can significantly compromise post-injury recovery and decrease quality of life. Identification of molecular mechanisms responsible for the breakdown in communication between immune and stress axes could highlight novel therapeutic targets to improve treatment after brain injury.

## Author Contributions

ZT: wrote the manuscript. JG: provided expertise in neuroinflammation and edited the manuscript. OK-C: provided expertise in TBI and edited the manuscript.

### Conflict of Interest Statement

The authors declare that the research was conducted in the absence of any commercial or financial relationships that could be construed as a potential conflict of interest.

## References

[1] Thurman DJ Alverson C Dunn KA Guerrero J Sniezek JE. Traumatic brain injury in the United States: a public health perspective. J Head Trauma Rehabil. (1999) 14:602–15. 10.1097/00001199-199912000-00009 10671706

[2] Fuller GW Ransom J Mandrekar J Brown AW. Long-term survival following traumatic brain injury: a population-based parametric survival analysis. Neuroepidemiology. (2016) 47:1–10. 10.1159/000445997 27165161 PMC5537588

[3] Polinder S Haagsma JA van Klaveren D Steyerberg EW van Beeck EF. Health-related quality of life after TBI: a systematic review of study design, instruments, measurement properties, and outcome. Popul Health Metr. (2015) 13:4. 10.1186/s12963-015-0037-1 25722656 PMC4342191

[4] Koponen S Taiminen T Portin R Himanen L Isoniemi H Heinonen H. Axis I and II psychiatric disorders after traumatic brain injury: a 30-year follow-up study. Am J Psychiatry. (2002) 159:1315–21. 10.1176/appi.ajp.159.8.1315 12153823

[5] Fleminger S Long-term psychiatric disorders after traumatic brain injury. Eur J Anaesthesiol. (2008) 25:123–30. 10.1017/S0265021507003250 18289429

[6] DiSabato DJ Quan N Godbout JP. Neuroinflammation: the devil is in the details. J Neurochem. (2016) 139:136–53. 10.1111/jnc.13607 26990767 PMC5025335

[7] Witcher KG Eiferman DS Godbout JP. Priming the inflammatory pump of the CNS after traumatic brain injury. Trends Neurosci. (2015) 38:609–20. 10.1016/j.tins.2015.08.002 26442695 PMC4617563

[8] Ramsay DS Woods SC. Clarifying the roles of homeostasis and allostasis in physiological regulation. Psychol Rev. (2014) 121:225–47. 10.1037/a0035942 24730599 PMC4166604

[9] Powner DJ Boccalandro C. Adrenal insufficiency following traumatic brain injury in adults. Curr Opin Crit Care. (2008) 14:163–6. 10.1097/MCC.0b013e3282f57528 18388678

[10] Pearn ML Niesman IR Egawa J Sawada A Almenar-Queralt A Shah SB. Pathophysiology associated with traumatic brain injury: current treatments and potential novel therapeutics. Cell Mol Neurobiol. (2017) 37:571–85. 10.1007/s10571-016-0400-1 27383839 PMC11482200

[11] Marklund N. Rodent models of traumatic brain injury: methods and challenges. Methods Mol Biol. (2016) 1462:29–46. 10.1007/978-1-4939-3816-2_3 27604711

[12] McIntosh TK Noble L Andrews B Faden AI. Traumatic brain injury in the rat: characterization of a midline fluid-percussion model. Cent Nerv Syst Trauma. (1987) 4:119–34. 10.1089/cns.1987.4.119 3690695

[13] Dixon CE Clifton GL Lighthall JW Yaghmai AA Hayes RL. A controlled cortical impact model of traumatic brain injury in the rat. J Neurosci Methods. (1991) 39:253–62. 1787745 10.1016/0165-0270(91)90104-8

[14] Chen H Chan YL Nguyen LT Mao Y de Rosa A Beh IT. Moderate traumatic brain injury is linked to acute behaviour deficits and long term mitochondrial alterations. Clin Exp Pharmacol Physiol. (2016) 43:1107–14. 10.1111/1440-1681.12650 27557565

[15] Wang L Wang L Dai Z Wu P Shi H Zhao S. Lack of mitochondrial ferritin aggravated neurological deficits via enhancing oxidative stress in a traumatic brain injury murine model. Biosci Rep. (2017) 37:BSR20170942. 10.1042/BSR20170942 28963372 PMC5672084

[16] Thelin EP Tajsic T Zeiler FA Menon DK Hutchinson PJA Carpenter KLH. Monitoring the neuroinflammatory response following acute brain injury. Front Neurol. (2017) 8:351. 10.3389/fneur.2017.00351 28775710 PMC5517395

[17] Kokiko-Cochran O Ransohoff L Veenstra M Lee S Saber M Sikora M. Altered neuroinflammation and behavior after traumatic brain injury in a mouse model of Alzheimer's disease. J Neurotrauma. (2016) 33:625–40. 26414955 10.1089/neu.2015.3970 PMC4971425

[18] Webster SJ Van Eldik LJ Watterson DM Bachstetter AD. Closed head injury in an age-related Alzheimer mouse model leads to an altered neuroinflammatory response and persistent cognitive impairment. J Neurosci. (2015) 35:6554–69. 10.1523/JNEUROSCI.0291-15.2015 25904805 PMC4405562

[19] Corrigan F Mander KA Leonard AV Vink R. Neurogenic inflammation after traumatic brain injury and its potentiation of classical inflammation. J Neuroinflammation. (2016) 13:264. 10.1186/s12974-016-0738-9 27724914 PMC5057243

[20] Chiu C-C Liao Y-E Yang L-Y Wang J-Y Tweedie D Karnati HK. Neuroinflammation in animal models of traumatic brain injury. J Neurosci Methods. (2016) 272:38–49. 10.1016/j.jneumeth.2016.06.018 27382003 PMC5201203

[21] Irvine K-A Bishop RK Won SJ Xu J Hamel KA Coppes V Effects of veliparib on microglial activation and functional outcomes after traumatic brain injury in the rat and pig. J Neurotrauma. (2018) 35:918–29. 10.1089/neu.2017.5044 29285982

[22] Fenn AM Skendelas JP Moussa DN Muccigrosso MM Popovich PG Lifshitz J. Methylene blue attenuates traumatic brain injury-associated neuroinflammation and acute depressive-like behavior in mice. J Neurotrauma. (2015) 32:127–38. 10.1089/neu.2014.3514 25070744 PMC4291210

[23] Kokiko-Cochran ON Saber M Puntambekar S Bemiller SM Katsumoto A Lee Y-S. Traumatic brain injury in hTau model mice: enhanced acute macrophage response and altered long-term recovery. J Neurotrauma. (2018) 35:73–84. 10.1089/neu.2017.5203 28859549 PMC5757085

[24] Cao T Thomas TC Ziebell JM Pauly JR Lifshitz J. Morphological and genetic activation of microglia after diffuse traumatic brain injury in the rat. Neuroscience. (2012) 225:65–75. 10.1016/j.neuroscience.2012.08.058 22960311 PMC3489473

[25] Brooks DM Patel SA Wohlgehagen ED Semmens EO Pearce A Sorich EA. Multiple mild traumatic brain injury in the rat produces persistent pathological alterations in the brain. Exp Neurol. (2017) 297:62–72. 10.1016/j.expneurol.2017.07.015 28756201

[26] Turtzo L Lescher J Janes L Dean DD Budde MD Frank JA. Macrophagic and microglial responses after focal traumatic brain injury in the female rat. J Neuroinflammation. (2014) 11:82. 10.1186/1742-2094-11-82 24761998 PMC4022366

[27] Kumar A Stoica BA Loane DJ Yang M Abulwerdi G Khan N. Microglial-derived microparticles mediate neuroinflammation after traumatic brain injury. J Neuroinflammation. (2017) 14:47. 10.1186/s12974-017-0819-4 28292310 PMC5351060

[28] Minogue AM Barrett JP Lynch MA. LPS-induced release of IL-6 from glia modulates production of IL-1β in a JAK2-dependent manner. J Neuroinflammation. (2012) 9:629. 10.1186/1742-2094-9-126 22697788 PMC3418561

[29] Witcher KG Bray CE Dziabis JE McKim DB Benner BN Rowe RK. Traumatic brain injury-induced neuronal damage in the somatosensory cortex causes formation of rod-shaped microglia that promote astrogliosis and persistent neuroinflammation. Glia. (2018) 66:2719–36. 10.1002/glia.23523 30378170 PMC7542609

[30] Loane DJ Kumar A Stoica BA Cabatbat R Faden AI. Progressive neurodegeneration after experimental brain trauma. J Neuropathol Exp Neurol. (2014) 73:14–29. 10.1097/NEN.0000000000000021 24335533 PMC4267248

[31] Neri M Frati A Turillazzi E Cantatore S Cipolloni L Di Paolo M. Immunohistochemical evaluation of aquaporin-4 and its correlation with CD68, IBA-1, HIF-1α, GFAP, and CD15 expressions in fatal traumatic brain injury. Int J Mol Sci. (2018) 19:3544. 10.3390/ijms19113544 30423808 PMC6274714

[32] Ramlackhansingh AF Brooks DJ Greenwood RJ Bose SK Turkheimer FE Kinnunen KM. Inflammation after trauma: Microglial activation and traumatic brain injury. Ann Neurol. (2011) 70:374–83. 10.1002/ana.22455 21710619

[33] Surace MJ Block ML. Targeting microglia-mediated neurotoxicity: the potential of NOX2 inhibitors. Cell Mol Life Sci. (2012) 69:2409–27. 10.1007/s00018-012-1015-4 22581365 PMC3677079

[34] Dohi K Ohtaki H Nakamachi T Yofu S Satoh K Miyamoto K. Gp91phox (NOX2) in classically activated microglia exacerbates traumatic brain injury. J Neuroinflammation. (2010) 7:41. 10.1186/1742-2094-7-41 20659322 PMC2917406

[35] Kumar A Barrett JP Alvarez-Croda D-M Stoica BA Faden AI Loane DJ. NOX2 drives M1-like microglial/macrophage activation and neurodegeneration following experimental traumatic brain injury. Brain Behav Immun. (2016) 58:291–309. 10.1016/j.bbi.2016.07.158 27477920 PMC5067217

[36] Schiavone S Neri M Trabace L Turillazzi E. The NADPH oxidase NOX2 mediates loss of parvalbumin interneurons in traumatic brain injury: human autoptic immunohistochemical evidence. Sci Rep. (2017) 7:8752. 10.1038/s41598-017-09202-4 28821783 PMC5562735

[37] Zanier ER Marchesi F Ortolano F Perego C Arabian M Zoerle T. Fractalkine receptor deficiency is associated with early protection but late worsening of outcome following brain trauma in mice. J Neurotrauma. (2016) 33:1060–72. 10.1089/neu.2015.4041 26180940 PMC4892197

[38] Elmore MRP Najafi AR Koike MA Dagher NN Spangenberg EE Rice RA. Colony-stimulating factor 1 receptor signaling is necessary for microglia viability, unmasking a microglia progenitor cell in the adult brain. Neuron. (2014) 82:380–97. 10.1016/j.neuron.2014.02.040 24742461 PMC4161285

[39] Yamasaki TR Blurton-Jones M Morrissette DA Kitazawa M Oddo S LaFerla FM. Neural stem cells improve memory in an inducible mouse model of neuronal loss. J Neurosci. (2007) 27:11925–33. 10.1523/JNEUROSCI.1627-07.2007 17978032 PMC6673368

[40] Rice RA Spangenberg EE Yamate-Morgan H Lee RJ Arora RPS Hernandez MX. Elimination of microglia improves functional outcomes following extensive neuronal loss in the hippocampus. J Neurosci. (2015) 35:9977–89. 10.1523/JNEUROSCI.0336-15.2015 26156998 PMC4495246

[41] Rice RA Pham J Lee RJ Najafi AR West BL Green KN. Microglial repopulation resolves inflammation and promotes brain recovery after injury. Glia. (2017) 65:931–44. 10.1002/glia.23135 28251674 PMC5395311

[42] Lull ME Block ML. Microglial activation and chronic neurodegeneration. Neurotherapeutics. (2010) 7:354–65. 10.1016/j.nurt.2010.05.014 20880500 PMC2951017

[43] Cunningham C Wilcockson DC Campion S Lunnon K Perry VH. Central and systemic endotoxin challenges exacerbate the local inflammatory response and increase neuronal death during chronic neurodegeneration. J Neurosci. (2005) 25:9275–84. 10.1523/JNEUROSCI.2614-05.2005 16207887 PMC6725757

[44] Norden DM Godbout JP. Review: microglia of the aged brain: primed to be activated and resistant to regulation. Neuropathol Appl Neurobiol. (2013) 39:19–34. 10.1111/j.1365-2990.2012.01306.x 23039106 PMC3553257

[45] Fenn AM Gensel JC Huang Y Popovich PG Lifshitz J Godbout JP. Immune activation promotes depression 1 month after diffuse brain injury: a role for primed microglia. Biol Psychiatry. (2014) 76:575–84. 10.1016/j.biopsych.2013.10.014 24289885 PMC4000292

[46] Torres-Platas SG Cruceanu C Chen GG Turecki G Mechawar N. Evidence for increased microglial priming and macrophage recruitment in the dorsal anterior cingulate white matter of depressed suicides. Brain Behav Immun. (2014) 42:50–9. 10.1016/j.bbi.2014.05.007 24858659

[47] Spencer RL Deak T. A users guide to HPA axis research. Physiol Behav. (2017) 178:43–65. 10.1016/j.physbeh.2016.11.014 27871862 PMC5451309

[48] Herman JP McKlveen JM Solomon MB Carvalho-Netto E Myers B. Neural regulation of the stress response: glucocorticoid feedback mechanisms. Brazilian J Med Biol Res. (2012) 45:292–8. 10.1590/S0100-879X2012007500041 22450375 PMC3854162

[49] Herman JP Dolgas CM Carlson SL. Ventral subiculum regulates hypothalamo-pituitary-adrenocortical and behavioural responses to cognitive stressors. Neuroscience. (1998) 86:449–59. 10.1016/S0306-4522(98)00055-4 9881860

[50] Diorio D Viau V Meaney MJ. The role of the medial prefrontal cortex (cingulate gyrus) in the regulation of hypothalamic-pituitary-adrenal responses to stress. J Neurosci. (1993) 13:3839–47. 10.1523/JNEUROSCI.13-09-03839.1993 8396170 PMC6576467

[51] Deng Q Riquelme D Trinh L Low MJ Tomić M Stojilkovic S Aguilera G. Rapid glucocorticoid feedback inhibition of ACTH secretion involves ligand-dependent membrane association of glucocorticoid receptors. Endocrinology. (2015) 156:3215–27. 10.1210/EN.2015-1265 26121342 PMC4541620

[52] Russell GM Henley DE Leendertz J Douthwaite JA Wood SA Stevens A. Rapid glucocorticoid receptor-mediated inhibition of hypothalamic-pituitary-adrenal ultradian activity in healthy males. J Neurosci. (2010) 30:6106–15. 10.1523/JNEUROSCI.5332-09.2010 20427668 PMC6632590

[53] Walker JJ Spiga F Gupta R Zhao Z Lightman SL Terry JR. Rapid intra-adrenal feedback regulation of glucocorticoid synthesis. J R Soc Interface. (2015) 12:20140875. 10.1098/rsif.2014.0875 25392395 PMC4277077

[54] Tasker JG. Rapid glucocorticoid actions in the hypothalamus as a mechanism of homeostatic integration. Obesity. (2006) 14:259S−65S. 10.1038/oby.2006.320 17021378

[55] Laryea G Arnett M Muglia LJ. Ontogeny of hypothalamic glucocorticoid receptor-mediated inhibition of the hypothalamic-pituitary-adrenal axis in mice. Stress. (2015) 18:400–7. 10.3109/10253890.2015.1046832 26068518 PMC5704948

[56] Aronsson M Fuxe K Dong Y Agnati LF Okret S Gustafsson JA. Localization of glucocorticoid receptor mRNA in the male rat brain by in situ hybridization. Proc Natl Acad Sci USA. (1988) 85:9331–5. 10.1073/pnas.85.23.9331 3194428 PMC282733

[57] Leon-Mercado L Herrera Moro Chao D Basualdo MDC Kawata M Escobar C Buijs RM. The arcuate nucleus: a site of fast negative feedback for corticosterone secretion in male rats. eNeuro. (2017) 4:1–14. 10.1523/ENEURO.0350-16.2017 28275717 PMC5334455

[58] Schulkin J Morgan MA Rosen JB. A neuroendocrine mechanism for sustaining fear. Trends Neurosci. (2005) 28:629–35. 10.1016/j.tins.2005.09.009 16214230

[59] Liberman AC Budziñski ML Sokn C Gobbini RP Steininger A Arzt E. Regulatory and mechanistic actions of glucocorticoids on T and inflammatory cells. Front Endocrinol. (2018) 9:235. 10.3389/fendo.2018.00235 29867767 PMC5964134

[60] Barnes PJ. Anti-inflammatory actions of glucocorticoids: molecular mechanisms. Clin Sci. (1998) 94:557–72. 9854452 10.1042/cs0940557

[61] Heitzer MD Wolf IM Sanchez ER Witchel SF DeFranco DB. Glucocorticoid receptor physiology. Rev Endocr Metab Disord. (2007) 8:321–30. 10.1007/s11154-007-9059-8 18049904

[62] Cruz-Topete D Cidlowski JA. One hormone, two actions: anti- and pro-inflammatory effects of glucocorticoids. Neuroimmunomodulation. (2015) 22:20–32. 10.1159/000362724 25227506 PMC4243162

[63] Sierra A Gottfried-Blackmore A Milner TA McEwen BS Bulloch K. Steroid hormone receptor expression and function in microglia. Glia. (2008) 56:659–74. 10.1002/glia.20644 18286612

[64] Golde S Coles A Lindquist JA Compston A. Decreased iNOS synthesis mediates dexamethasone-induced protection of neurons from inflammatory injury *in vitro*. Eur J Neurosci. (2003) 18:2527–37. 10.1046/j.1460-9568.2003.02917.x 14622153

[65] Luísa Vital A Gonç alo MC Teresa Cruz M rico Figueiredo A Duarte CB Celeste Lopes M Dexamethasone prevents granulocyte-macrophage colony-stimulating factor-induced nuclear factor-kB activation, inducible nitric oxide synthase expression and nitric oxide production in a skin dendritic cell line. Mediators Inflamm. (2003) 12:71–8. 10.1080/0962935031000097673 12775356 PMC1781603

[66] Herold MJ McPherson KG Reichardt HM. Glucocorticoids in T cell apoptosis and function. Cell Mol Life Sci. (2006) 63:60–72. 10.1007/s00018-005-5390-y 16314919 PMC2792342

[67] Salvador E Shityakov S Förster C. Glucocorticoids and endothelial cell barrier function. Cell Tissue Res. (2014) 355:597–605. 10.1007/s00441-013-1762-z 24352805 PMC3972429

[68] Hue CD Cho FS Cao S Dale Bass CR Meaney DF Morrison B. Dexamethasone potentiates *in vitro* blood-brain barrier recovery after primary blast injury by glucocorticoid receptor-mediated upregulation of ZO-1 tight junction protein. J Cereb Blood Flow Metab. (2015) 35:1191–8. 10.1038/jcbfm.2015.38 25757751 PMC4640274

[69] Sorrells SF Sapolsky RM. An inflammatory review of glucocorticoid actions in the CNS. Brain Behav Immun. (2007) 21:259–72. 10.1016/j.bbi.2006.11.006 17194565 PMC1997278

[70] Bergold PJ. Treatment of traumatic brain injury with anti-inflammatory drugs. Exp Neurol. (2016) 275:367–80. 10.1016/j.expneurol.2015.05.024 26112314 PMC6007860

[71] Zhang Z Zhang Z Artelt M Burnet M Schluesener HJ. Dexamethasone attenuates early expression of three molecules associated with microglia/macrophages activation following rat traumatic brain injury. Acta Neuropathol. (2007) 113:675–82. 10.1007/s00401-007-0195-8 17265048

[72] Roberts I Yates D Sandercock P Farrell B Wasserberg J Lomas G Effect of intravenous corticosteroids on death within 14 days in 10 008 adults with clinically significant head injury (MRC CRASH trial): randomised placebo-controlled trial. Lancet. (2004) 364:1321–8. 10.1016/S0140-6736(04)17188-2 15474134

[73] Barnes PJ Adcock IM. Glucocorticoid resistance in inflammatory diseases. Lancet. (2009) 373:1905–17. 10.1016/S0140-6736(09)60326-3 19482216

[74] Sapolsky RM Krey LC McEwen BS. The neuroendocrinology of stress and aging: the glucocorticoid cascade hypothesis. Endocr Rev. (1986) 7:284–301. 10.1210/edrv-7-3-284 3527687

[75] Frank MG Miguel ZD Watkins LR Maier SF. Prior exposure to glucocorticoids sensitizes the neuroinflammatory and peripheral inflammatory responses to *E. coli* lipopolysaccharide. Brain Behav Immun. (2010) 24:19–30. 10.1016/j.bbi.2009.07.008 19647070

[76] Frank MG Thompson BM Watkins LR Maier SF. Glucocorticoids mediate stress-induced priming of microglial pro-inflammatory responses. Brain Behav Immun. (2012) 26:337–45. 10.1016/j.bbi.2011.10.005 22041296 PMC5652300

[77] McCullers DL Sullivan PG Scheff SW Herman JP. Mifepristone protects CA1 hippocampal neurons following traumatic brain injury in rat. Neuroscience. (2002) 109:219–30. 10.1016/S0306-4522(01)00477-8 11801359

[78] Calcia MA Bonsall DR Bloomfield PS Selvaraj S Barichello T Howes OD. Stress and neuroinflammation: a systematic review of the effects of stress on microglia and the implications for mental illness. Psychopharmacology. (2016) 233:1637–50. 10.1007/s00213-016-4218-9 26847047 PMC4828495

[79] Wiegers GJ Labeur MS Stec IE Klinkert WE Holsboer F Reul JM. Glucocorticoids accelerate anti-T cell receptor-induced T cell growth. J Immunol. (1995) 155:1893–902. 7636240

[80] Niraula A Wang Y Godbout JP Sheridan JF. Corticosterone production during repeated social defeat causes monocyte mobilization from the bone marrow, glucocorticoid resistance, and neurovascular adhesion molecule expression. J Neurosci. (2018) 38:2328–40. 10.1523/JNEUROSCI.2568-17.2018 29382712 PMC5830519

[81] Weber MD Godbout JP Sheridan JF. Repeated social defeat, neuroinflammation and behavior: monocytes carry the signal. Neuropsychopharmacology. (2017) 42:46–61. 10.1038/npp.2016.102 27319971 PMC5143478

[82] MacPherson A Dinkel K Sapolsky R. Glucocorticoids worsen excitotoxin-induced expression of pro-inflammatory cytokines in hippocampal cultures. Exp Neurol. (2005) 194:376–83. 10.1016/j.expneurol.2005.02.021 16022865

[83] Marsland AL Walsh C Lockwood K John-Henderson NA. The effects of acute psychological stress on circulating and stimulated inflammatory markers: a systematic review and meta-analysis. Brain Behav Immun. (2017) 64:208–19. 10.1016/j.bbi.2017.01.011 28089638 PMC5553449

[84] Ruzek MC Pearce BD Miller AH Biron CA. Endogenous glucocorticoids protect against cytokine-mediated lethality during viral infection. J Immunol. (1999) 162:3527–33. 10092810

[85] Tursich M Neufeld RWJ Frewen PA Harricharan S Kibler JL Rhind SG. Association of trauma exposure with proinflammatory activity: a transdiagnostic meta-analysis. Transl Psychiatry. (2014) 4:e413. 10.1038/tp.2014.56 25050993 PMC4119223

[86] Cleare AJ Miell J Heap E Sookdeo S Young L Malhi GS. Hypothalamo-pituitary-adrenal axis dysfunction in chronic fatigue syndrome, and the effects of low-dose hydrocortisone therapy. J Clin Endocrinol Metab. (2001) 86:3545–54. 10.1210/jcem.86.8.7735 11502777

[87] Komaroff AL. Inflammation correlates with symptoms in chronic fatigue syndrome. Proc Natl Acad Sci USA. (2017) 114:8914–6. 10.1073/pnas.1712475114 28811366 PMC5576849

[88] Zuckerman A Ram O Ifergane G Matar MA Kaplan Z Hoffman JR. Role of endogenous and exogenous corticosterone on behavioral and cognitive responses to low-pressure blast wave exposure. J Neurotrauma. (2018) 36:380–94. 10.1089/neu.2018.5672 29947272

[89] Cernak I Savic VJ Lazarov A Joksimovic M Markovic S. Neuroendocrine responses following graded traumatic brain injury in male adults. Brain Inj. (1999) 13:1005–15. 10628505 10.1080/026990599121016

[90] Taylor AN Rahman SU Tio DL Sanders MJ Bando JK Truong AH. Lasting neuroendocrine-immune effects of traumatic brain injury in rats. J Neurotrauma. (2006) 23:1802–13. 10.1089/neu.2006.23.1802 17184190

[91] Taylor AN Rahman SU Sanders NC Tio DL Prolo P Sutton RL. Injury severity differentially affects short- and long-term neuroendocrine outcomes of traumatic brain injury. J Neurotrauma. (2008) 25:311–23. 10.1089/neu.2007.0486 18373481

[92] Bernard F Outtrim J Menon DK Matta BF. Incidence of adrenal insufficiency after severe traumatic brain injury varies according to definition used: clinical implications. Br J Anaesth. (2006) 96:72–6. 10.1093/bja/aei277 16311283

[93] Aimaretti G Ambrosio MR Di Somma C Fusco A Cannavo S Gasperi M. Traumatic brain injury and subarachnoid haemorrhage are conditions at high risk for hypopituitarism: screening study at 3 months after the brain injury. Clin Endocrinol. (2004) 61:320–6. 10.1111/j.1365-2265.2004.02094.x 15355447

[94] Lieberman SA Oberoi AL Gilkison CR Masel BE Urban RJ Prevalence of neuroendocrine dysfunction in patients recovering from traumatic brain injury^1^. J Clin Endocrinol Metab. (2001) 86:2752–6. 10.1210/jcem.86.6.7592 11397882

[95] Kokshoorn NE Wassenaar MJE Biermasz NR Roelfsema F Smit JWA Romijn JA. Hypopituitarism following traumatic brain injury: prevalence is affected by the use of different dynamic tests and different normal values. Eur J Endocrinol. (2010) 162:11–8. 10.1530/EJE-09-0601 19783619

[96] Rowe RK Rumney BM May HG Permana P Adelson PD Harman SM. Diffuse traumatic brain injury affects chronic corticosterone function in the rat. Endocr Connect. (2016) 5:152–66. 10.1530/EC-16-0031 27317610 PMC5002959

[97] Osterstock G El Yandouzi T Romanò N Carmignac D Langlet F Coutry N. Sustained alterations of hypothalamic tanycytes during posttraumatic hypopituitarism in male mice. Endocrinology. (2014) 155:1887–98. 10.1210/en.2013-1336 24601879

[98] Dennis EL Faskowitz J Rashid F Babikian T Mink R Babbitt C. Diverging volumetric trajectories following pediatric traumatic brain injury. NeuroImage Clin. (2017) 15:125–35. 10.1016/j.nicl.2017.03.014 28507895 PMC5423316

[99] Tölli A Borg J Bellander B-M Johansson F Höybye C Pituitary function within the first year after traumatic brain injury or subarachnoid haemorrhage. J Endocrinol Invest. (2017) 40:193–205. 10.1007/s40618-016-0546-1 27671168 PMC5269462

[100] Webster JB Bell KR. Primary adrenal insufficiency following traumatic brain injury: a case report and review of the literature. Arch Phys Med Rehabil. (1997) 78:314–8. 9084356 10.1016/s0003-9993(97)90040-x

[101] Xiong Y Mahmood A Chopp M. Current understanding of neuroinflammation after traumatic brain injury and cell-based therapeutic opportunities. Chinese J Traumatol. (2018) 21:137–51. 10.1016/j.cjtee.2018.02.003 29764704 PMC6034172

[102] Grundy PL Harbuz MS Jessop DS Lightman SL Sharples PM. The hypothalamo-pituitary-adrenal axis response to experimental traumatic brain injury. J Neurotrauma. (2001) 18:1373–81. 10.1089/08977150152725669 11780867

[103] Roe SY McGowan EM Rothwell NJ. Evidence for the involvement of corticotrophin-releasing hormone in the pathogenesis of traumatic brain injury. Eur J Neurosci. (1998) 10:553–9. 10.1046/j.1460-9568.1998.00064.x 9749718

[104] Hannon MJ Crowley RK Behan LA O'Sullivan EP O'Brien MMC Sherlock M. Acute glucocorticoid deficiency and diabetes insipidus are common after acute traumatic brain injury and predict mortality. J Clin Endocrinol Metab. (2013) 98:3229–37. 10.1210/jc.2013-1555 23690314

[105] Tanriverdi F De Bellis A Ulutabanca H Bizzarro A Sinisi AA Bellastella G. A five year prospective investigation of anterior pituitary function after traumatic brain injury: is hypopituitarism long-term after head trauma associated with autoimmunity? J Neurotrauma. (2013) 30:1426–33. 10.1089/neu.2012.2752 23470214

[106] Ewing-Cobbs L Prasad MR Cox CS Granger DA Duque G Swank PR. Altered stress system reactivity after pediatric injury: Relation with post-traumatic stress symptoms. Psychoneuroendocrinology. (2017) 84:66–75. 10.1016/j.psyneuen.2017.06.003 28667938 PMC5555029

[107] Niederland T Makovi H Gál V Andréka B Ábrahám CS Kovács J. Abnormalities of pituitary function after traumatic brain injury in children. J Neurotrauma. (2007) 24:119–27. 10.1089/neu.2005.369ER 17263675

[108] van Bodegom M Homberg JR Henckens MJAG. Modulation of the hypothalamic-pituitary-adrenal axis by early life stress exposure. Front Cell Neurosci. (2017) 11:87. 10.3389/fncel.2017.00087 28469557 PMC5395581

[109] Erlanger DM Kutner KC Jacobs AR. Hormones and cognition: current concepts and issues in neuropsychology. Neuropsychol Rev. (1999) 9:175–207. 10.1023/A:1021634622577 10667447

[110] Murphy FC Michael A Robbins TW Sahakian BJ. Neuropsychological impairment in patients with major depressive disorder: the effects of feedback on task performance. Psychol Med. (2003) 33:455–67. 10.1017/S0033291702007018 12701666

[111] Keller J Gomez R Williams G Lembke A Lazzeroni L Murphy GM. HPA axis in major depression: cortisol, clinical symptomatology and genetic variation predict cognition. Mol Psychiatry. (2017) 22:527–36. 10.1038/mp.2016.120 27528460 PMC5313380

[112] Broshek DK De Marco AP Freeman JR. A review of post-concussion syndrome and psychological factors associated with concussion. Brain Inj. (2015) 29:228–37. 10.3109/02699052.2014.974674 25383595

[113] Zihl J Almeida OFX. Neuropsychology of neuroendocrine dysregulation after traumatic brain injury. J Clin Med. (2015) 4:1051–62. 10.3390/jcm4051051 26239465 PMC4470216

[114] Molaie AM Maguire J. Neuroendocrine abnormalities following traumatic brain injury: an important contributor to neuropsychiatric sequelae. Front Endocrinol. (2018) 9:176. 10.3389/fendo.2018.00176 29922224 PMC5996920

[115] West TA Sharp S. Neuroendocrine dysfunction following mild TBI: when to screen for it. J Fam Pract. (2014) 63:11–6. 24475461

[116] Voorhees JL Tarr AJ Wohleb ES Godbout JP Mo X Sheridan JF. Prolonged restraint stress increases IL-6, reduces IL-10, and causes persistent depressive-like behavior that is reversed by recombinant IL-10. PLoS ONE. (2013) 8:e58488. 10.1371/journal.pone.0058488 23520517 PMC3592793

[117] Sathyanesan M Haiar JM Watt MJ Newton SS. Restraint stress differentially regulates inflammation and glutamate receptor gene expression in the hippocampus of C57BL/6 and BALB/c mice. Stress. (2017) 20:197–204. 10.1080/10253890.2017.1298587 28274152 PMC5724770

[118] Yanagita S Amemiya S Suzuki S Kita I. Effects of spontaneous and forced running on activation of hypothalamic corticotropin-releasing hormone neurons in rats. Life Sci. (2007) 80:356–63. 10.1016/j.lfs.2006.09.027 17067638

[119] Svensson M Rosvall P Boza-Serrano A Andersson E Lexell J Deierborg T. Forced treadmill exercise can induce stress and increase neuronal damage in a mouse model of global cerebral ischemia. Neurobiol Stress. (2016) 5:8–18. 10.1016/j.ynstr.2016.09.002 27981192 PMC5145912

[120] Griesbach GS Tio DL Vincelli J McArthur DL Taylor AN. Differential effects of voluntary and forced exercise on stress responses after traumatic brain injury. J Neurotrauma. (2012) 29:1426–33. 10.1089/neu.2011.2229 22233388 PMC3335105

[121] Jutkiewicz EM Wood SK Houshyar H Hsin L-W Rice KC Woods JH. The effects of CRF antagonists, antalarmin, CP154,526, LWH234, and R121919, in the forced swim test and on swim-induced increases in adrenocorticotropin in rats. Psychopharmacology. (2005) 180:215–23. 10.1007/s00213-005-2164-z 15696320 PMC1315297

[122] Torner L Plotsky PM Neumann ID de Jong TR. Forced swimming-induced oxytocin release into blood and brain: Effects of adrenalectomy and corticosterone treatment. Psychoneuroendocrinology. (2017) 77:165–74. 10.1016/j.psyneuen.2016.12.006 28064086

[123] Gagliano H Ortega-Sanchez JA Nadal R Armario A. Psychostimulants and forced swim stress interaction: how activation of the hypothalamic-pituitary-adrenal axis and stress-induced hyperglycemia are affected. Psychopharmacology. (2017) 234:2859–69. 10.1007/s00213-017-4675-9 28710520

[124] de Kloet ER Molendijk ML. Coping with the forced swim stressor: towards understanding an adaptive mechanism. Neural Plast. (2016) 2016:1–13. 10.1155/2016/6503162 27034848 PMC4806646

[125] Lovelock DF Deak T. Repeated exposure to two stressors in sequence demonstrates that corticosterone and paraventricular nucleus of the hypothalamus interleukin-1β responses habituate independently. J Neuroendocrinol. (2017) 29:1–15. 10.1111/jne.12514 28803453 PMC5617797

[126] Cordero MI Merino JJ Sandi C. Correlational relationship between shock intensity and corticosterone secretion on the establishment and subsequent expression of contextual fear conditioning. Behav Neurosci. (1998) 112:885–91. 10.1037/0735-7044.112.4.885 9733194

[127] Blandino P Barnum CJ Solomon LG Larish Y Lankow BS Deak T. Gene expression changes in the hypothalamus provide evidence for regionally-selective changes in IL-1 and microglial markers after acute stress. Brain Behav Immun. (2009) 23:958–68. 10.1016/j.bbi.2009.04.013 19464360

[128] Reger ML Poulos AM Buen F Giza CC Hovda DA Fanselow MS. Concussive brain injury enhances fear learning and excitatory processes in the amygdala. Biol Psychiatry. (2012) 71:335–43. 10.1016/j.biopsych.2011.11.007 22169439 PMC3264758

[129] Han KS Kim L Shim I. Stress and sleep disorder. Exp Neurobiol. (2012) 21:141–50. 10.5607/en.2012.21.4.141 23319874 PMC3538178

[130] Toth LA Bhargava P. Animal models of sleep disorders. Comp Med. (2013) 63:91–104. 23582416 PMC3625050

[131] Paterson LM Nutt DJ Wilson SJ. Sleep and its disorders in translational medicine. J Psychopharmacol. (2011) 25:1226–34. 10.1177/0269881111400643 21490119

[132] Born J Späth-Schwalbe E Schwakenhofer H Kern W Fehm HL. Influences of corticotropin-releasing hormone, adrenocorticotropin, and cortisol on sleep in normal man. J Clin Endocrinol Metab. (1989) 68:904–11. 2541159 10.1210/jcem-68-5-904

[133] Steiger A. Neurochemical regulation of sleep. J Psychiatr Res. (2007) 41:537–52. 10.1016/j.jpsychires.2006.04.007 16777143

[134] Buckley TM Schatzberg AF. On the interactions of the Hypothalamic-Pituitary-Adrenal (HPA) axis and sleep: normal HPA axis activity and circadian rhythm, exemplary sleep disorders. J Clin Endocrinol Metab. (2005) 90:3106–14. 10.1210/jc.2004-1056 15728214

[135] Guyon A Morselli LL Balbo ML Tasali E Leproult R L'Hermite-Balériaux M. Effects of insufficient sleep on pituitary-adrenocortical response to CRH stimulation in healthy men. Sleep. (2017) 40:1–10. 10.1093/sleep/zsx064 28444400 PMC6075556

[136] Rampin C Cespuglio R Chastrette N Jouvet M. Immobilisation stress induces a paradoxical sleep rebound in rat. Neurosci Lett. (1991) 126:113–8. 10.1016/0304-3940(91)90532-X 1922920

[137] van Dalfsen JH Markus CR. The influence of sleep on human hypothalamic–pituitary–adrenal (HPA) axis reactivity: a systematic review. Sleep Med Rev. (2018) 39:187–94. 10.1016/j.smrv.2017.10.002 29126903

[138] Weibel L Follenius M Spiegel K Ehrhart J Brandenberger G. Comparative effect of night and daytime sleep on the 24-hour cortisol secretory profile. Sleep. (1995) 18:549–56. 8552925

[139] Sauvet F Florence G Van Beers P Drogou C Lagrume C Chaumes C. Total sleep deprivation alters endothelial function in rats: a nonsympathetic mechanism. Sleep. (2014) 37:465–73. 10.5665/sleep.3476 24587568 PMC3920311

[140] Kelliher P Connor TJ Harkin A Sanchez C Kelly JP Leonard BE. Varying responses to the rat forced-swim test under diurnal and nocturnal conditions. Physiol Behav. (2000) 69:531–9. 10.1016/S0031-9384(00)00213-4 10913793

[141] Pawlyk AC Jha SK Brennan FX Morrison AR Ross RJ. A rodent model of sleep disturbances in posttraumatic stress disorder: the role of context after fear conditioning. Biol Psychiatry. (2005) 57:268–77. 10.1016/j.biopsych.2004.11.008 15691528

[142] Coenen AM van Luijtelaar EL. Stress induced by three procedures of deprivation of paradoxical sleep. Physiol Behav. (1985) 35:501–4. 4070421 10.1016/0031-9384(85)90130-1

[143] Yehuda S Sredni B Carasso RL Kenigsbuch-Sredni D. REM sleep deprivation in rats results in inflammation and interleukin-17 elevation. J Interf Cytokine Res. (2009) 29:393–8. 10.1089/jir.2008.0080 19450150

[144] Medeiros R Lenneberg-Hoshino C Hoshino K Tufik S. Neuroethologic differences in sleep deprivation induced by the single- and multiple-platform methods. Brazilian J Med Biol Res. (1998) 31:675–80. 10.1590/S0100-879X1998000500012 9698774

[145] Vecsey CG Baillie GS Jaganath D Havekes R Daniels A Wimmer M. Sleep deprivation impairs cAMP signalling in the hippocampus. Nature. (2009) 461:1122–5. 10.1038/nature08488 19847264 PMC2783639

[146] Sorge RE Martin LJ Isbester KA Sotocinal SG Rosen S Tuttle AH. Olfactory exposure to males, including men, causes stress and related analgesia in rodents. Nat Methods. (2014) 11:629–32. 10.1038/nmeth.2935 24776635

[147] Gross BA Vanderheyden WM Urpa LM Davis DE Fitzpatrick CJ Prabhu K. Stress-free automatic sleep deprivation using air puffs. J Neurosci Methods. (2015) 251:83–91. 10.1016/j.jneumeth.2015.05.010 26014662 PMC4589302

[148] He J Hsuchou H He Y Kastin AJ Wang Y Pan W. Sleep restriction impairs blood-brain barrier function. J Neurosci. (2014) 34:14697–706. 10.1523/JNEUROSCI.2111-14.2014 25355222 PMC4212067

[149] Viola-Saltzman M Musleh C. Traumatic brain injury-induced sleep disorders. Neuropsychiatr Dis Treat. (2016) 12:339. 10.2147/NDT.S69105 26929626 PMC4760657

[150] Rowe RK Harrison JL Morrison H Subbian V Murphy SM Lifshitz J Acute post-traumatic sleep may define vulnerability to a second traumatic brain injury in mice. J Neurotrauma. (2018) 35:1–17. 10.1089/neu.2018.5980 30398389 PMC6479254

[151] Rowe RK Harrison JL O'Hara BF Lifshitz J Diffuse brain injury does not affect chronic sleep patterns in the mouse. Brain Inj. (2014) 28:504–10. 10.3109/02699052.2014.888768 24702469 PMC7482552

[152] Inutsuka A Yamanaka A. The physiological role of orexin/hypocretin neurons in the regulation of sleep/wakefulness and neuroendocrine functions. Front Endocrinol. (2013) 4:18. 10.3389/fendo.2013.00018 23508038 PMC3589707

[153] Thomasy HE Opp MR. Hypocretin mediates sleep and wake disturbances in a mouse model of traumatic brain injury. J Neurotrauma. (2019) 36:802–14. 10.1089/neu.2018.5810 30136622 PMC6387567

[154] Haghighi F Ge Y Chen S Xin Y Umali MU De Gasperi R. Neuronal DNA methylation profiling of blast-related traumatic brain injury. J Neurotrauma. (2015) 32:1200–9. 10.1089/neu.2014.3640 25594545 PMC4532898

[155] Mullington JM Simpson NS Meier-Ewert HK Haack M. Sleep loss and inflammation. Best Pract Res Clin Endocrinol Metab. (2010) 24:775–84. 10.1016/j.beem.2010.08.014 21112025 PMC3548567

[156] Stephenson R Caron A Sleep deprivation does not affect neuronal susceptibility to mild traumatic brain injury in the rat. Nat Sci Sleep. (2015) 7:63 10.2147/NSS.S82888 26124685 PMC4482367

[157] Rowe RK Harrison JL O'Hara BF Lifshitz J. Recovery of neurological function despite immediate sleep disruption following diffuse brain injury in the mouse: clinical relevance to medically untreated concussion. Sleep. (2014) 37:743–52. 10.5665/sleep.3582 24899763 PMC4044747

[158] Macera CA Aralis HJ Rauh MJ MacGregor AJ. Do sleep problems mediate the relationship between traumatic brain injury and development of mental health symptoms after deployment? Sleep. (2013) 36:83–90. 10.5665/sleep.2306 23288974 PMC3524546

[159] Mollayeva T Shapiro CM Mollayeva S Cassidy JD Colantonio A. Modeling community integration in workers with delayed recovery from mild traumatic brain injury. BMC Neurol. (2015) 15:194. 10.1186/s12883-015-0432-z 26452471 PMC4600293

[160] Gilbert KS Kark SM Gehrman P Bogdanova Y. Sleep disturbances, TBI and PTSD: Implications for treatment and recovery. Clin Psychol Rev. (2015) 40:195–212. 10.1016/j.cpr.2015.05.008 26164549 PMC5153364

[161] Grandhi R Tavakoli S Ortega C Simmonds M A review of chronic pain and cognitive, mood, and motor dysfunction following mild traumatic brain injury: complex, comorbid, and/or overlapping conditions? Brain Sci. (2017) 7:160 10.3390/brainsci7120160 PMC5742763 29211026

[162] Mollayeva T D'Souza A Mollayeva S. Sleep and psychiatric disorders in persons with mild traumatic brain injury. Curr Psychiatry Rep. (2017) 19:47. 10.1007/s11920-017-0800-z 28653116

[163] Cheng W Rolls ET Ruan H Feng J. Functional connectivities in the brain that mediate the association between depressive problems and sleep quality. JAMA Psychiatry. (2018) 75:1052. 10.1001/jamapsychiatry.2018.1941 30046833 PMC6233808

[164] Stuss DT. Traumatic brain injury: relation to executive dysfunction and the frontal lobes. Curr Opin Neurol. (2011) 24:584–9. 21968550 10.1097/WCO.0b013e32834c7eb9

[165] Merkley TL Larson MJ Bigler ED Good DA Perlstein WM. Structural and functional changes of the cingulate gyrus following traumatic brain injury: relation to attention and executive skills. J Int Neuropsychol Soc. (2013) 19:899–910. 10.1017/S135561771300074X 23845701

[166] Borniger JC Ungerleider K Zhang N Karelina K Magalang UJ Weil ZM. Repetitive brain injury of juvenile mice impairs environmental enrichment-induced modulation of REM sleep in adulthood. Neuroscience. (2018) 375:74–83. 10.1016/j.neuroscience.2018.01.064 29432885 PMC6668616

[167] Blinman TA Houseknecht E Snyder C Wiebe DJ Nance ML. Postconcussive symptoms in hospitalized pediatric patients after mild traumatic brain injury. J Pediatr Surg. (2009) 44:1223–8. 10.1016/j.jpedsurg.2009.02.027 19524745

[168] Tham SW Palermo TM Vavilala MS Wang J Jaffe KM Koepsell TD. The longitudinal course, risk factors, and impact of sleep disturbances in children with traumatic brain injury. J Neurotrauma. (2012) 29:154–61. 10.1089/neu.2011.2126 22029569 PMC3253307

[169] Morse A Garner D. Traumatic brain injury, sleep disorders, and psychiatric disorders: an underrecognized relationship. Med Sci. (2018) 6:15. 10.3390/medsci6010015 29462866 PMC5872172

[170] Kinder HA Baker EW West FD. The pig as a preclinical traumatic brain injury model: current models, functional outcome measures, and translational detection strategies. Neural Regen Res. (2019) 14:413–24. 10.4103/1673-5374.245334 30539807 PMC6334610

[171] Manchester K Corrigan JD Singichetti B Huang L Bogner J Yi H. Current health status and history of traumatic brain injury among Ohio adults. Inj Prev. (2019). 10.1136/injuryprev-2018-043056. [Epub ahead of print]. 30803993

[172] Martinez BI Stabenfeldt SE. Current trends in biomarker discovery and analysis tools for traumatic brain injury. J Biol Eng. (2019) 13:16. 10.1186/s13036-019-0145-8 30828380 PMC6381710

[173] Mohamadpour M Whitney K Bergold PJ. The importance of therapeutic time window in the treatment of traumatic brain injury. Front Neurosci. (2019) 13:07. 10.3389/fnins.2019.00007 30728762 PMC6351484

[174] Epelbaum J Vrontakis M Magnaghi V Quinn M Agha A Post-Traumatic hypopituitarism-who should be screened, when, and how? Front Endocrinol. (2018) 9:8 10.3389/fendo.2018.00008 PMC5801312 29456522

